# Optimal doses of different exercise types for improving depressive symptoms in climacteric women: a systematic review and network meta-analysis

**DOI:** 10.3389/fpsyg.2026.1727315

**Published:** 2026-04-07

**Authors:** Hongyu Wang, Shuang Li, Shicheng Cui, Yanqiong Wang, Ying Zhu, Rujia Zhou, Zihao Zhong, Xiaolin Zhang, Zixian Xiao, Qian Qin, Jiacheng Feng, Pengfei Wang, Dong Li

**Affiliations:** 1 College of Physical Education and Health, Guangxi Normal University, Guilin, China; 2 Taylor's University, Kuala Lumpur, Selangor, Malaysia; 3 School of Physical Education and Health, Zhaoqing University, Zhaoqing, China; 4 Department of Physical Education, Tianjin University of Technology, Tianjin, China; 5 Department of Sports and Leisure, Dongshin University, Naju-si, Republic of Korea

**Keywords:** climacteric women, depressive, dose, exercise, network

## Abstract

**Background:**

Depression in climacteric women—defined as females transitioning from perimenopause to postmenopause (perimenopause: declining ovarian function with fluctuating and decreasing estrogen levels; postmenopause: cessation of menstruation for ≥12 months with persistently low estrogen levels)—impairs quality of life and burdens families and healthcare systems. Exercise, a proven non-pharmacological intervention for menopausal depression, has been understudied for “optimal dosage across exercise types”—previous research either focused on activity-depression associations or ignored dosage when selecting exercise types. This systematic review and network meta-analysis addresses this gap by comparing optimal doses of four exercises (aerobic, multi-mode, stretching, and mind–body) to establish an evidence-based “exercise type-dose” hierarchy for clinical practice.

**Methods:**

We systematically searched PubMed, Cochrane Library, Embase, and Web of Science (inception to September 2025) for randomized controlled trials (RCTs) on exercise for climacteric women’s depression. Two researchers independently screened studies, extracted data, and assessed quality via Cochrane RoB2. Bayesian network meta-regression (MBNMA, R software) modeled dose–response relationships, and SUCRA ranked intervention efficacy to identify optimal doses.

**Results:**

Twenty-three RCTs were included. The most favorable ranges observed in available RCTs appear to be: aerobic exercise (600–1,100 MET-min/week, slight saturation); multi-mode exercise (1000–1,500 MET-min/week); stretching exercise (900–1,200 MET-min/week, U-shaped curve); mind–body exercise (1000–1,500 MET-min/week, limited data >1,500 MET-min/week). Three sessions/week and 12-week intervention aligned with these most favorable ranges.

**Conclusion:**

Aerobic (600–1,100 MET-min/week), multi-mode (1000–1,500 MET-min/week), and stretching (900–1,200 MET-min/week) exercises are most effective for alleviating climacteric depression. Clinicians should prioritize aerobic exercise; multi-mode exercise may improve adherence for those seeking variety. Key challenges include supplementing high-dose mind–body exercise data (>1,500 MET-min/week) and supporting long-term adherence amid menopausal physiological changes.

**Systematic review registration:**

This study has been registered on PROSPERO (CRD420251162965).

## 1 Introduction

Menopausal depression in climacteric women refers to the mental disorder emerging during the transition from perimenopause to postmenopause, posing a significant threat to women’s physical and mental health globally (Zender and Olshansky, 2009; Clayton and Ninan, 2010). Its pathogenesis is closely linked to sharp declines in estrogen levels leading to neuroendocrine dysregulation (Soares, 2023), with an incidence of 25.99%, a threefold increase compared to non-climacteric populations (Li et al., 2016), driven by hormonal fluctuations, aging, social stressors, and genetic susceptibility (Li et al., 2016; Woods and Mitchell, 2005). Characterized by depressive mood, anxiety, sleep disturbances, and cognitive decline (Llaneza et al., 2012), the condition severely impairs quality of life and social functioning, with heightened disability burdens in regions with cultural constraints and societal pressures (Palacios et al., 2010), making targeted prevention and early intervention a global public health priority (Bagga et al., 2024).

In the management of climacteric depression, pharmacological interventions (e.g., neurotransmitter modulators, hormone replacement therapy) have limited applicability due to individual response variations (Frey et al., 2008; Klaiber et al., 1996), potential risks in specific populations (Nelson et al., 2002), and suboptimal efficacy for hormone-related mood disorders and somatic.

symptoms (Whedon et al., 2017; Santoro et al., 2015). Given the core pathogenesis involves multidimensional imbalance of the “endocrine-neuropsychological-social” system (Bagga et al., 2024; Best, 2008; Simpson et al., 2025), non-pharmacological interventions have emerged as a valuable alternative.

Exercise, a well-established non-pharmacological approach, effectively alleviates depressive symptoms in this population (Lialy et al., 2023), with various modalities (e.g., running, Pilates, and Baduanjin) concurrently regulating hormone levels, improving sleep quality, and enhancing psychological resilience (Liu et al., 2023; Sternfeld and Dugan, 2011; Yue et al., 2025). These interventions offer advantages such as low adverse reactions, high cost-effectiveness, and good adherence (Mendoza et al., 2016; Elavsky and McAuley, 2007). Critical to exercise efficacy and safety is dosage—encompassing frequency, intensity, duration per session, and total intervention period (Hansford et al., 2022; Gallois et al., 2017). For climacteric women, suboptimal dosage (too low to activate neuroendocrine regulation; too high to burden the vulnerable musculoskeletal system or induce emotional rebound) undermines outcomes (Morss et al., 2004; Izquierdo et al., 2021), highlighting the need for further exploration of dosage ranges tailored to individual characteristics (Tang et al., 2024; McInnis and Morehead, 2020). However, existing evidence on optimal dosages across different exercise types remains limited: prior meta-analyses have primarily focused on exercise-depression associations (Yue et al., 2025; Liu and Tang, 2025), while one network meta-analysis identified suitable exercise types but overlooked dosage considerations (Wang et al., 2025), leaving a gap in evidence to guide targeted exercise prescriptions.

To address this evidence gap and generate hypotheses about potential effective dose ranges, this study conducted a systematic review and network meta-analysis of high-quality RCTs. Its objective is to explore the comparative effectiveness of different exercise types and their associated dose–response patterns, with the aim of identifying preliminary, evidence-informed dose ranges for clinical reference. Ultimately, this exploratory analysis seeks to answer the research question: What are the potential favorable dose ranges for different exercise types in alleviating climacteric depression?

## 2 Methods

### 2.1 Protocol and registration

This systematic review was carried out in strict compliance with the Preferred Reporting Items for Systematic Reviews and Meta-Analyses (PRISMA) 2020 guidelines, and followed all specifications for study inclusion criteria, data organization processes, statistical analysis approaches, and result reporting standards. The study protocol was prospectively registered with the International Prospective Register of Systematic Reviews (PROSPERO), and assigned the unique registration number CRD420251162965.

### 2.2 Data sources and search strategy

A comprehensive systematic search was conducted across four electronic databases (PubMed, Cochrane Library, Embase, and Web of Science) to identify literature on various exercise interventions for climacteric women. To ensure complete coverage, the reference lists of eligible studies were screened for additional publications. The search period encompassed records from the inception of the databases up to September 4, 2025. The search strategy was developed according to the PICOS criteria (Population: climacteric women; Intervention: physical exercise; Comparison: any control; Outcome: depression indicators; Study design: randomized controlled trials). The main search terms included: “Exercises” or “Physical Activity” or “Activities, Physical” or “Physical Activities” or “Exercise, Physical” or “Physical Exercise” and “Depression” or “Depressive Symptom” or “Emotional Depression” or “Negative Emotion” or “Affective Disorder” and “climacteric women” or “menopause” or “postmenopause” or “climacteric” or “menopausal” or “perimenopausal” or “menopausal woman” For detailed search strategies, please consult Appendices B, B1.

### 2.3 Study selection

After applying the above search strategy, two researchers (HYW and DL) independently screened studies per the PRISMA guidelines. Preliminary screening entailed title/abstract reviews to spot potentially qualified studies using pre-established inclusion criteria. Articles that met initial screening thresholds were subject to full-text retrieval and rigorous evaluation. For quantitative synthesis, the final decision on study inclusion was made via discussion and consensus—any discrepancies were settled through team discussions until a consensus was reached.

### 2.4 Inclusion and exclusion criteria

The systematic review was based on the PICOS framework to establish the study selection criteria.

Studies that met all of the following criteria were included:

1. Study design: Randomized controlled trials with parallel-group or crossover designs.
2. Participants: Climacteric women (perimenopausal or postmenopausal as defined) with depressive symptoms confirmed by validated scales, regardless of whether they had a formal clinical diagnosis of depressive disorder. Participants with subthreshold depression, mild depressive symptoms, or climacteric symptoms measured by scales incorporating mood-related items were also eligible.
3. Intervention: Structured physical activity/exercise programs with clear frequency, intensity, duration, and mode specifications. Multi-mode exercise refers to combinations of multiple physical exercise forms (no non-exercise components); combined interventions with non-exercise components are eligible only if a separate exercise-only control group is set to isolate exercise effects.
4. Outcomes: Depression severity data measured before and after the intervention using validated scales.
5. Data completeness: Sufficient original or extractable data (mean, standard deviation, sample size, precise *p*-values) to calculate effect sizes (e.g., standardized mean differences).
6. Language: Full-text articles published in English.

Studies were excluded if they met any of the following criteria:

1. Study design: Observational studies (e.g., cross-sectional studies, case–control studies, cohort studies).
2. Participants: Studies involving non-climacteric women, including those with undetermined menopausal status, chemotherapy/radiotherapy-induced menopause, or participants aged < 40 years.
3. Intervention: Exercise interventions with insufficient program details; combined interventions with non-exercise components where exercise effects cannot be separated; multi-mode exercise containing non-exercise components.
4. Study type: Qualitative studies, reviews, theses, or conference abstracts.
5. Data incompleteness: Missing key outcome data or non-extractable data (e.g., descriptive statistics without numerical values).
6. Ethical issues: Violation of ethical standards (e.g., lack of informed consent, disproportionate risk–benefit ratio).
7. Language: Non-English publications.

### 2.5 Data extraction

To guarantee the dependability of the literature retrieval and screening process, once the search was finalized, two researchers (HYW and DL) independently assessed the titles, abstracts, and full texts of the retrieved studies. Inter rater reliability across the two screening stages was subsequently measured via Cohen’s kappa, encompassing two stages: the initial screening (based on title and abstract reviews) and the subsequent full-text screening. Consistency levels were classified as follows: fair (0.40–0.59), good (0.60–0.74), and excellent (>0.75) (Sim and Wright, 2005).

This systematic review followed the PICOS framework to establish the literature selection, inclusion, and exclusion criteria. Two researchers independently conducted data extraction using a standardized protocol. Any discrepancies during the process were resolved through consensus discussions with the research team. The data extracted from each included study covered the following aspects:

1. Basic study information: First author, publication year, country, and study design.
2. Demographic and clinical characteristics of participants: Sample size, mean age, population, diagnostic criteria, and medication usage.
3. Exercise-related variables: Type of intervention, intensity, frequency, total duration, single session duration, relevant metabolic equivalents (METs) and their corresponding codes.
4. Outcome measures: Depression severity data measured before and after the intervention using validated scales. For studies reporting multiple depressive outcomes (e.g., different scales or subscales of the same scale), priority was given to the primary outcome’s depression scale; if no primary outcome was specified, the most widely used or comprehensively scored depression scale was selected for analysis.

For studies that provided intervention parameters or outcome data in graphical form (with no numerical details), Engauge Digitizer software (v12.1) was employed to extract data accurately. In trials involving multiple follow-up evaluations, only data collected immediately after the intervention were included—this standardized temporal comparability across studies. If standard deviations (SDs) were unreported, they were computed using a formula derived from the 95% confidence interval (CI) of each group’s mean.

Furthermore, the exercise dose was represented in terms of task metabolic equivalent (MET), a physiological metric for evaluating energy expenditure during physical activity. MET is defined as the ratio of an individual’s exercise metabolic rate to their resting metabolic rate (Norton et al., 2010). When the initial studies did not report exercise intensity, we estimated it based on the type of exercise. The total dose of exercise was expressed as “MET-minutes/week,” calculated as the product of the intervention’s duration, frequency, and intensity. During the analysis, exercise dose was stratified in 200 MET increments, with groups including 200, 500, 750, 1,000, and 2000 METs. This stratification method aids in enhancing the stability and interpretability of the network meta-analysis results (Higgins et al., 2012).

### 2.6 Quality assessment

We used the Cochrane RoB2 assessment tool to evaluate the quality of studies across five domains: (1) randomization process, (2) deviations from intended interventions, (3) missing outcome data, (4) outcome measurement, and (5) selection of reported results. Based on these criteria, the overall risk of bias for each study was systematically assessed and categorized into three levels: low risk, high risk, or some concerns.

### 2.7 Statistical analysis

Meta-analysis (MBNMA) to explore the nonlinear dose–response relationship between different exercise types and doses in climacteric women. Before formal modeling, the analyzability of the network structure was first evaluated, with a focus on verifying assumptions of connectivity, transitivity, and consistency. To standardize results across different depression scales (e.g., GDS, BDI, and HADS) with varying scoring systems, intervention effects were measured using standardized mean differences (SMD) and their 95% credible intervals (CrIs), enabling quantitative synthesis and comparison across studies.

Dose–response curves were initially plotted based on observed trends; this was followed by fitting multiple nonlinear functional models (such as the Emax model, restricted cubic splines, quadratic polynomials, and non-monotonic growth curves) to capture potential underlying relationships. The optimal fitting function was identified by comparing 32 indicators—including the Deviance Information Criterion (DIC), model complexity, residual distribution, and heterogeneity metrics. After evaluation, the quadratic function consistently performed best across all indicators and was therefore selected as the final model for depicting the dose–response relationship.

All analyses were conducted in R (v4.4.2): the “MBNMAdose” package was used for model construction and parameter estimation, while the “ggplot2” package handled curve visualization. When exercise intensity was not explicitly reported in the included studies, MET values were estimated following a strict standardized process to ensure objectivity and reproducibility. Specifically, these values were derived based on the authoritative standards outlined in the 2024 Adult Compendium of Physical Activities (Herrmann et al., 2024) and the Position Statement on Physical Activity and Exercise Intensity Terminology (Norton et al., 2010), integrated with the specific forms of each exercise type. All MET value assignments avoided subjective judgments, with priority given to the intervals explicitly stated in authoritative guidelines. For complex or unique exercises lacking direct corresponding standards, MET values were derived using the approach of “referencing similar exercises + matching intensity descriptions.” The entire derivation logic was documented to ensure reproducibility and effectively mitigate selection bias. For acute exercise interventions, energy expenditure was calculated by multiplying the assigned MET value by the duration of each training session. For sustained interventions, weekly energy expenditure was determined by multiplying per-session duration by frequency, yielding an indicator expressed as MET-minutes per week. Continuous exercise dose data (MET-min/week) were divided into discrete categories to support network meta-analysis and improve statistical stability. The “three-arm design” refers to selecting three core dose categories (low: 250 MET-min/week, moderate: 1000 MET-min/week, high: 2000 MET-min/week) as the primary analysis framework.

To support network meta-analysis and improve statistical stability, continuous energy expenditure data were divided into discrete dose categories. The three-arm design strategically balanced methodological simplicity with robust variance estimation, enabling rigorous synthesis of heterogeneous evidence networks without compromising result interpretability. To assess the effectiveness of different interventions, surface under the cumulative ranking curve (SUCRA) values were calculated, and results were presented in probability ranking tables.

Sensitivity analysis: Given that some included trials primarily targeted climacteric symptoms (e.g., hot flashes, sleep disturbances) with scales that incorporate mood items (rather than focusing exclusively on depression), a sensitivity analysis was conducted. This analysis excluded trials where depressive symptoms were not the primary outcome or where mood items accounted for <30% of the scale’s total score, to verify the robustness of the primary dose–response results.

## 3 Results

### 3.1 Trial selection

At the initial stage of literature search, researchers conducted a comprehensive search across four electronic databases, with a time span from the establishment of each database to September 4, 2025, and a total of 3,129 relevant studies were identified. After excluding 462 duplicate studies, the remaining 2,667 studies proceeded to the next screening stage; following initial screening based on titles and abstracts, 2,542 studies were excluded for failing to meet the inclusion criteria, and 125 studies finally entered the full-text review stage. At this stage, the inter-rater reliability between the two assessors reached a “good” level, with a Cohen’s kappa coefficient of 0.71 (a value ≥ 0.6 indicates substantial agreement, and this consistency is crucial for reducing subjective bias in screening).

**Figure 1:**
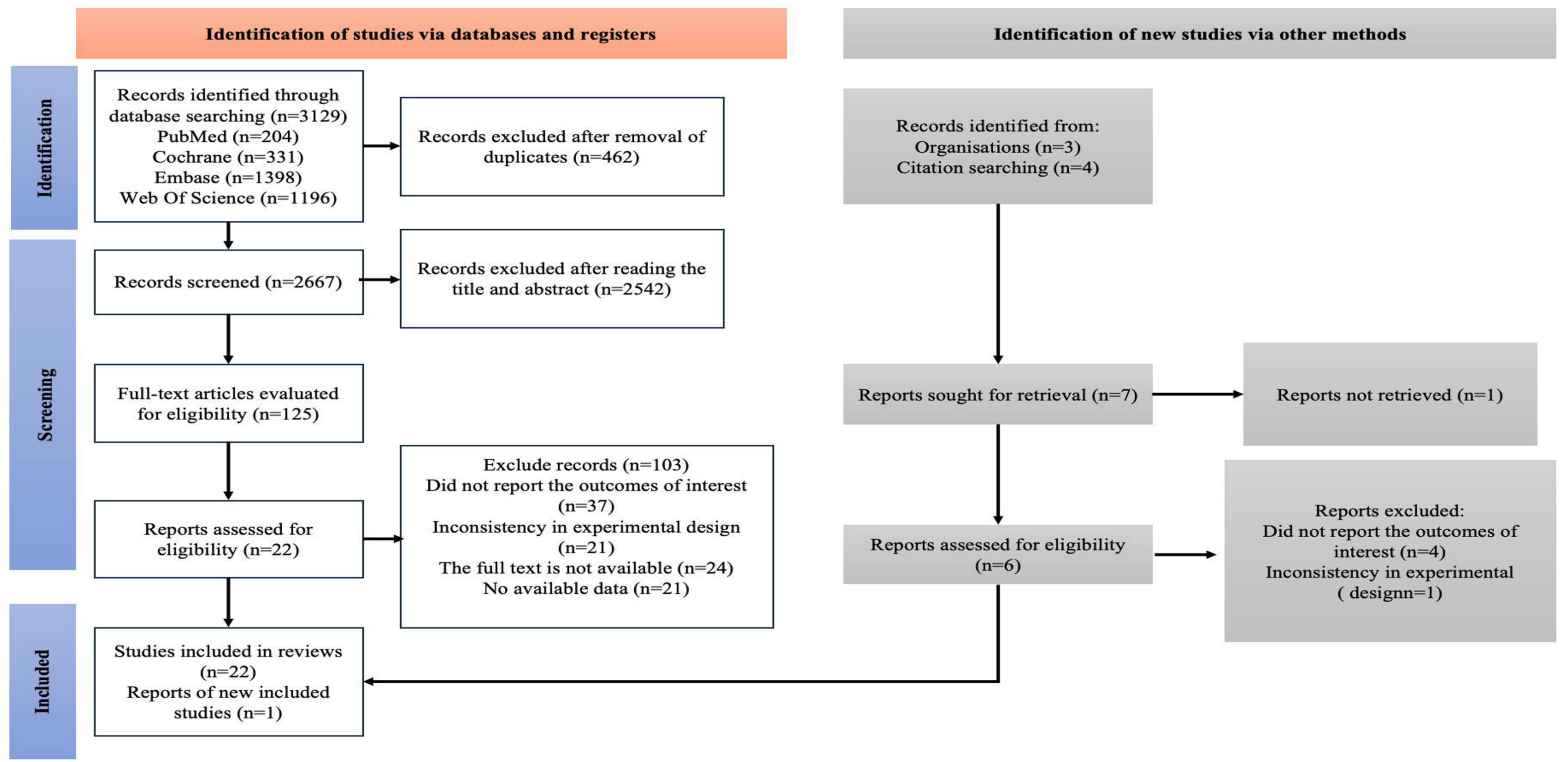
PRISMA flow diagram of the study process.

After full-text review, 103 studies were further excluded, with specific reasons including: unreported study results (*n* = 37), inconsistent experimental design (*n* = 21), unavailable full texts (*n* = 24), and lack of usable data (*n* = 21). The initial search ultimately identified 22 eligible studies; researchers further traced the reference lists of these studies and additionally identified 1 study that met the criteria (Figure 1). At this point, the inter-rater reliability between the two assessors improved to an “excellent” level, with a Cohen’s kappa coefficient of 0.81 (a value > 0.8 represents near-perfect consensus, which further enhanced the credibility of the finally included studies).

### 3.2 Trial characteristics

This review includes 23 randomized controlled trials published between 2006 and 2025 (Elavsky and McAuley, 2007; Pang and Kim, 2021; Bowen et al., 2006; Imayama et al., 2011; Bernard et al., 2015; Hu et al., 2017; Sternfeld et al., 2014; Abedi et al., 2015; Elsayed et al., 2022; Luoto et al., 2012; Noh et al., 2020; Sen et al., 2020; Aibar-Almazán et al., 2019; Abdoshahi, 2023; Afonso et al., 2012; Li et al., 2022; Newton et al., 2014; del Carmen Carcelén-Fraile et al., 2022; Arslan Kabasakal, 2025; Gao et al., 2016; Kai et al., 2016; Villaverde Gutierrez et al., 2012; Takahashi et al., 2019), with sample sizes in the intervention groups ranging from 13 to 117 participants, totaling 1,202 climacteric women. In the control groups, sample sizes ranged from 13 to 142 participants, with a total of 953 climacteric women. In both groups, the average age of the women exceeded 45 years.

Drawing on prior research, the interventions were grouped into four categories (Yue et al., 2025; Liu and Tang, 2025; Wang et al., 2025). Aerobic exercise, examined in 12 studies, relies on rhythmic engagement of major muscle groups to boost heart rate and breathing, in turn improving cardiovascular performance (e.g., walking, running, and cycling) (Elavsky and McAuley, 2007; Pang and Kim, 2021; Bowen et al., 2006; Imayama et al., 2011; Bernard et al., 2015; Hu et al., 2017; Sternfeld et al., 2014; Abedi et al., 2015; Elsayed et al., 2022; Luoto et al., 2012; Noh et al., 2020; Sen et al., 2020). Mind–body exercises, covered in 8 studies, are defined by integrating physical movement with mental adjustment, with a focus on harmonizing the body and mind. Common examples include yoga and Pilates, which prioritize breathing, posture, meditation, and relaxation methods to foster bodily and mental balance (Elavsky and McAuley, 2007; Aibar-Almazán et al., 2019; Abdoshahi, 2023; Afonso et al., 2012; Li et al., 2022; Newton et al., 2014; del Carmen Carcelén-Fraile et al., 2022; Arslan Kabasakal, 2025). Stretching exercises, analyzed in 3 studies, focus mainly on enhancing flexibility, correcting posture, and relieving muscle tightness through stretching different muscle groups. They often use static or dynamic stretches for specific body areas to loosen muscles and expand joint mobility (Afonso et al., 2012; Gao et al., 2016; Kai et al., 2016). Multi-mode motion, investigated in 4 studies, merges multiple exercise forms (e.g., aerobic and stretching workouts) into an integrated program intended to boost overall physical health and fitness via varied training approaches (Pang and Kim, 2021; Sen et al., 2020; Villaverde Gutierrez et al., 2012; Takahashi et al., 2019). All included studies utilized validated psychological assessment tools to evaluate depressive symptoms, including the Geriatric Depression Scale (GDS), Hospital Anxiety and Depression Scale (HADS), Depression Anxiety Stress Scale-21 (DASS-21), Greene Climacteric Scale (GCS), Brief Symptom Inventory (BSI), Brief Symptom Inventory-18 (BSI-18), and the Beck Depression Inventory (Table 1).

**Table 1:** Summary table of included reviews.

Study	Country	N (IG; CG)	Age (IG; CG)	Intervention (IG)			Intervention (CG)			Mets	Outcome measures
				Intervention content	Intervention time, frequency, period	Type	Intervention content	Intervention time, frequency, period	Type		
Villaverde Gutierrez et al. (2012)	Spain	30;30	60–70	Program of physical exercise	50–60 min, 2–3 weekly 6 months	Multi-mode motion	NI	NR	NI	IG:1000	GDS
Elavsky and McAuley (2007)	American	IG1:63; 39 IG2:62; 39	42–58	IG1: Walking IG2: Yoga	IG1:60 min 3 weekly 4 months IG2:90 min 2 weekly 4 months	IG1: Aerobic exercise IG2: Mind–body exercise	NI	NR	NI	IG1:750 IG2:500	GCS
Aibar-Almazán et al. (2019)	Spain	55; 55	69.15 ± 8.94; 69.15 ± 8.94	Pilates	60 min, 2 weekly, 12 weeks	Mind–body exercise	NI	NR	NI	IG:200	HADS
Pang and Kim (2021)	Korea	IG1:23; 16 IG2:13; 16	60.89 ± 6.62; 59.33 ± 6.54	IG1: Calendar training and exercise IG2: Exercise	12 weeks	IG1: Multi-mode motion IG2: Aerobic exercise	NI	NR	NI	IG1:1000 IG2:1000	GDS
Abdoshahi (2023)	Iran	16; 16	50–55	Pilates	2 weekly 3 mouths	Mind–body exercise	NI	NR	NI	IG1:200	DASS-21
Masaki Takahashi et al. (2019)	Singapore	19; 19	70.2 ± 3.9	Daily physical activity	75–150 min, 3–5 weekly, 8 weeks	Multi-mode motion	NI	NR	NI	IG1:1000	GDS
Bowen et al. (2006)	American	86; 86	50–75	Moderate-to-vigorous intensity aerobic exercise	45 min 5 weekly 12 months	Aerobic exercise	NI	NR	NI	IG1:2000	BSI
Imayama et al. (2011)	American	117; 87	50–75	Moderate-to-vigorous intensity aerobic exercise	45 min 5 weekly 12 months	Aerobic exercise	NI	NR	NI	IG1:2000	BSI-18
Afonso et al. (2012)	Brazil	IG1:14; 15 IG2:15; 15	50–65	IG1: Passive stretching IG2: Yoga	60 min 2 weekly 4 months	IG1: Stretching exercise IG2: Mind–body exercise	NI	NR	NI	IG1:200 IG2:1000	BDI
Bernard et al. (2015)	France	61; 60	57–75	Moderate intensity walking	45 min 3 weekly 6 months	Aerobic exercise	NI	NR	NI	IG1:500	BDI
Gao et al. (2016)	China	32；28	45–55	Square dance exercise	60–90 min, 5 weekly, 3 mouths	Stretching exercise	NI	NR	NI	IG1:2000	SDS
Hu et al., 2017	China	40; 40	45–65	Walking	4 mouths	Aerobic exercise	NI	NR	NI	IG1:500	BDI
Li et al. (2022)	Poland	17; 15	57.94(9.38); 58.23(11.81)	Baduanjin exercise	8 weeks	Mind–body exercise	NI	NR	NI	IG1:750	SDS
Sternfeld et al. (2014)	American	106; 142	40–62	Exercise program	3 weekly, 12 weeks	Aerobic exercise	NI	NR	NI	IG1:1000	PHQ-8
Newton et al. (2014)	American	107; 142	40–62	Yoga	90 min, 12 weekly, 12 weeks	Mind–body exercise	NI	NR	NI	IG1:200	PHQ-8
Abedi et al. (2015)	Iran	53; 53	52.4 ± 3.8; 53 ± 4.1	Walking	7 weekly, 12 weeks	Aerobic exercise	NI	NR	NI	IG1:1000	BDI
Elsayed et al. (2022)	Egypt	30; 30	58.79 ± 2.81; 58.79 ± 2.81	Aerobic exercise	3 weekly, 12 weeks	Aerobic exercise	NI	NR	NI	IG1:750	SDS
del Carmen Carcelén-Fraile et al. (2022)	Spain	63; 62	69.70 ± 6.15; 69.75 ± 6.76	Qigong	60 min, 2 weekly, 12 weeks	Mind–body exercise	NI	NR	NI	IG1:500	HADS
Arslan Kabasakal (2025)	Turkey	13; 13	59.45 ± 11.52; 59.45 ± 11.52	Pilates	60 min, 2 weekly, 6 weeks	Mind–body exercise	NI	NR	NI	IG1:200	BDI-PC
Luoto et al. (2012)	Finland	88; 88	54.5 ± 3.8; 54.2 ± 3.7	Aerobic exercise	50 min, 4 weekly, 6 mouths	Aerobic exercise	NI	NR	NI	IG1:1000	WHQ
Kai et al. (2016)	Japan	20; 20	51.0 ± 7.0; 51.2 ± 7.9	Stretching exercise	10 min, 7 weekly, 3 weeks	Stretching exercise	NI	NR	NI	IG1:200	SDS
Noh et al. (2020)	Korea	21; 19	50–65	Walking	60 min, 3 weekly, 12 weeks	Aerobic exercise	NI	NR	NI	IG1:750	SCL-95-R
Sen et al. (2020)	Turkey	IG1:38; 20 IG2:38; 20	40–65	IG1: Wbv IG2: High-intensity exercise	IG1:20–40 min, 3 weekly, 24 weeks IG2:10–30 min, 5 weekly, 12 weeks	IG1: Multi-mode motion IG2: Aerobic exercise	NI	NR	NI	IG1:2000 IG2:2000	BDI

IG, intervention group; CG, control group; N, number; NA: not available; NR, no report; NI, no intervention; GDS, Geriatric Depression Scale; HADS: Hospital Anxiety and Depression Scale; DASS-21: Depression Anxiety Stress Scale-21; GCS: Greene Climacteric Scale; BSI: Brief Symptom Inventory; BSI-18: Brief Symptom Inventory-18; BDI: Beck Depression Inventory; SDS: Self-Rating Depression Scale; PHQ-8: Patient Health Questionnaire-8; BDI-PC: Beck Depression Inventory for Primary Care; WHQ: Women’s Health Questionnaire; SCL-95-R: Korea Symptom-Checklist-95-Revision.

### 3.3 Risk of bias and evidence assessment

**Figure 2:**
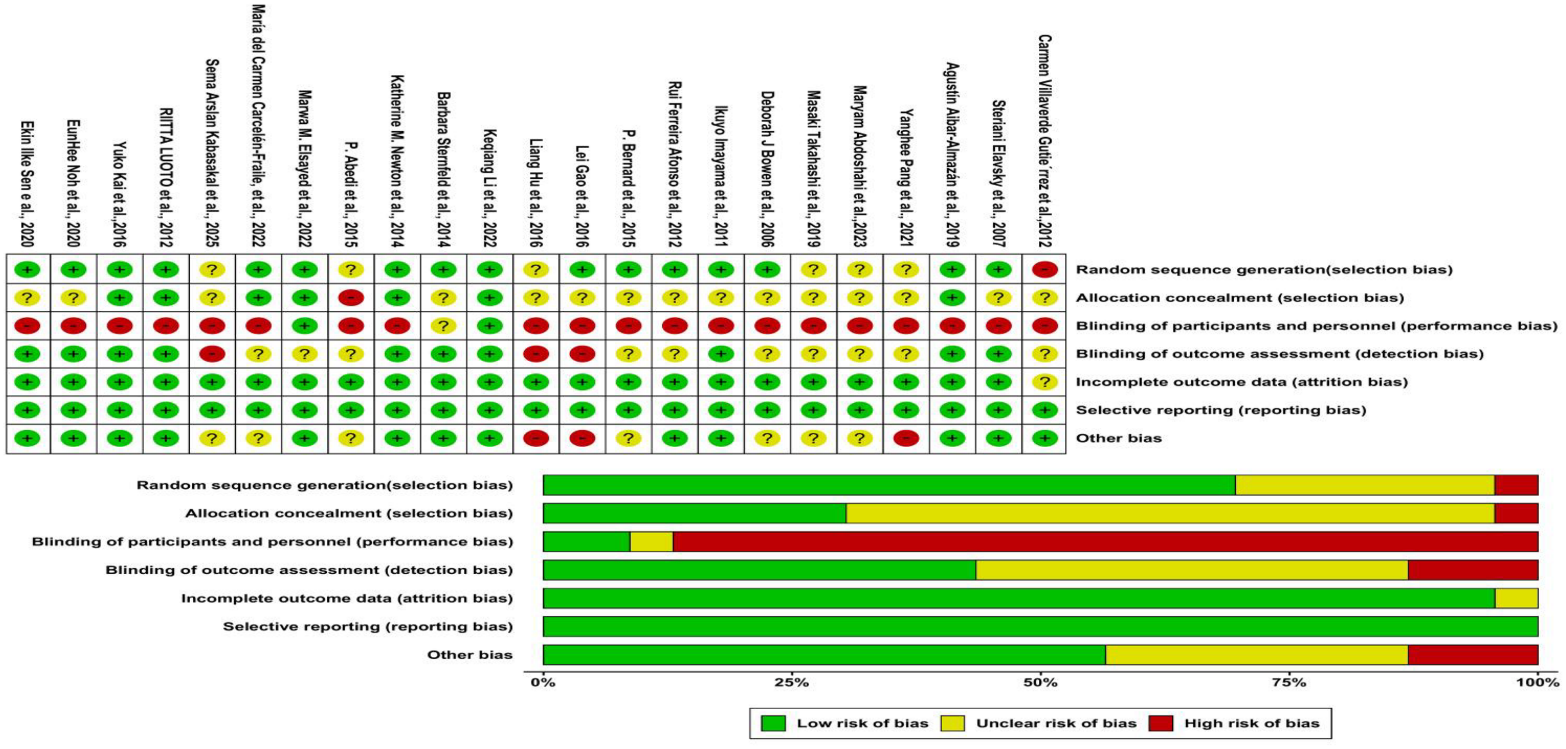
Risk of bias of included studies.

Of the 23 included studies, 16 were judged to have a low risk of bias in their randomization process, one was categorized as high risk, and six failed to provide specific details about their randomization methods. As for bias in outcome measurement, 22 studies were evaluated as low risk, whereas one was classified as high risk. With respect to selective reporting bias, all 23 studies were determined to be low risk, and none were found to be high risk. When evaluating the 23 studies against the five established criteria, their overall risk of bias was grouped as follows: 14 were identified as having an overall low risk, six as overall high risk, and three as having “some concerns.” A comprehensive overview of these bias risk assessments is provided in Figure 2.

### 3.4 Network meta-analysis of dose response

**Figure 3:**
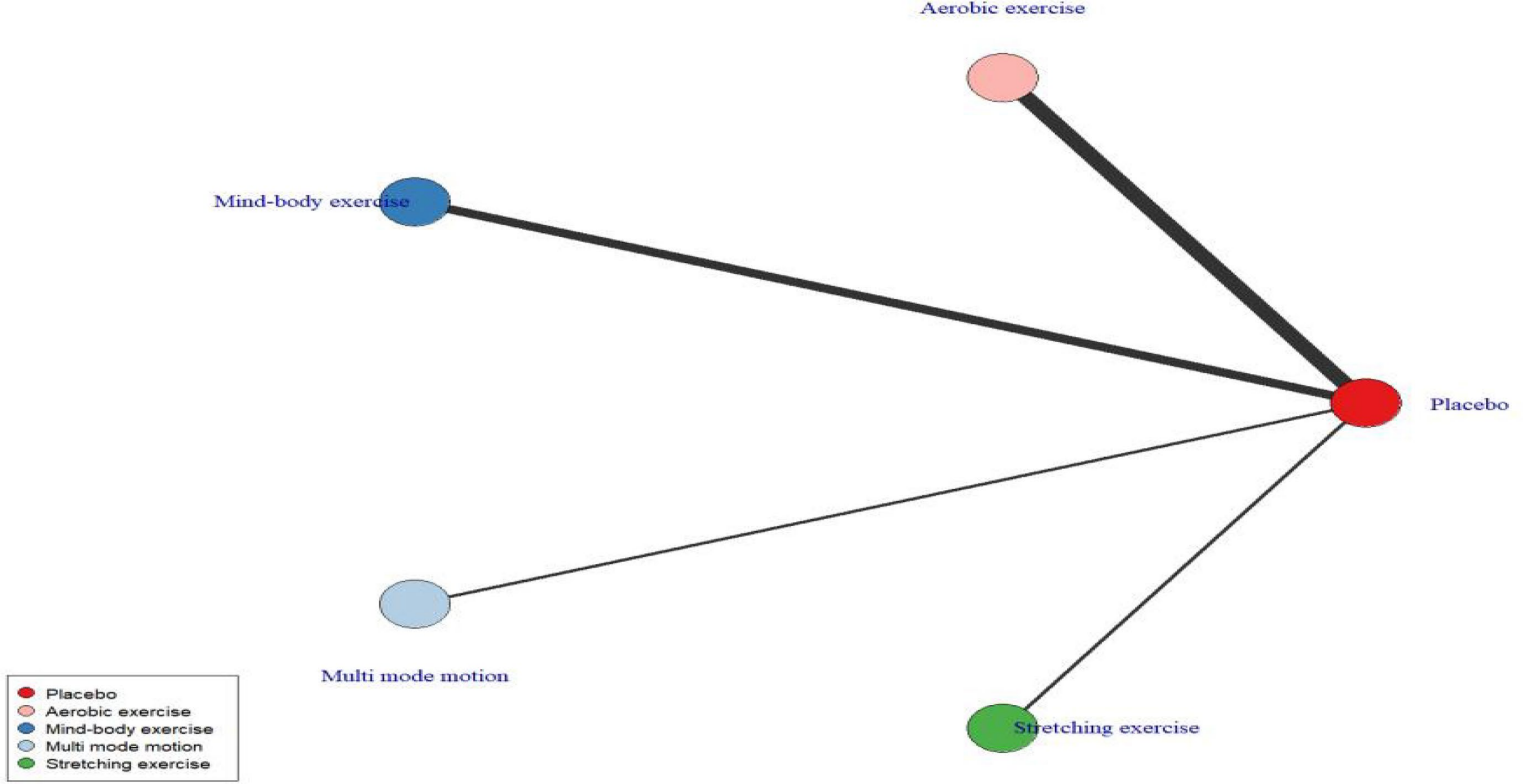
Exercise type network diagram.

**Figure 4:**
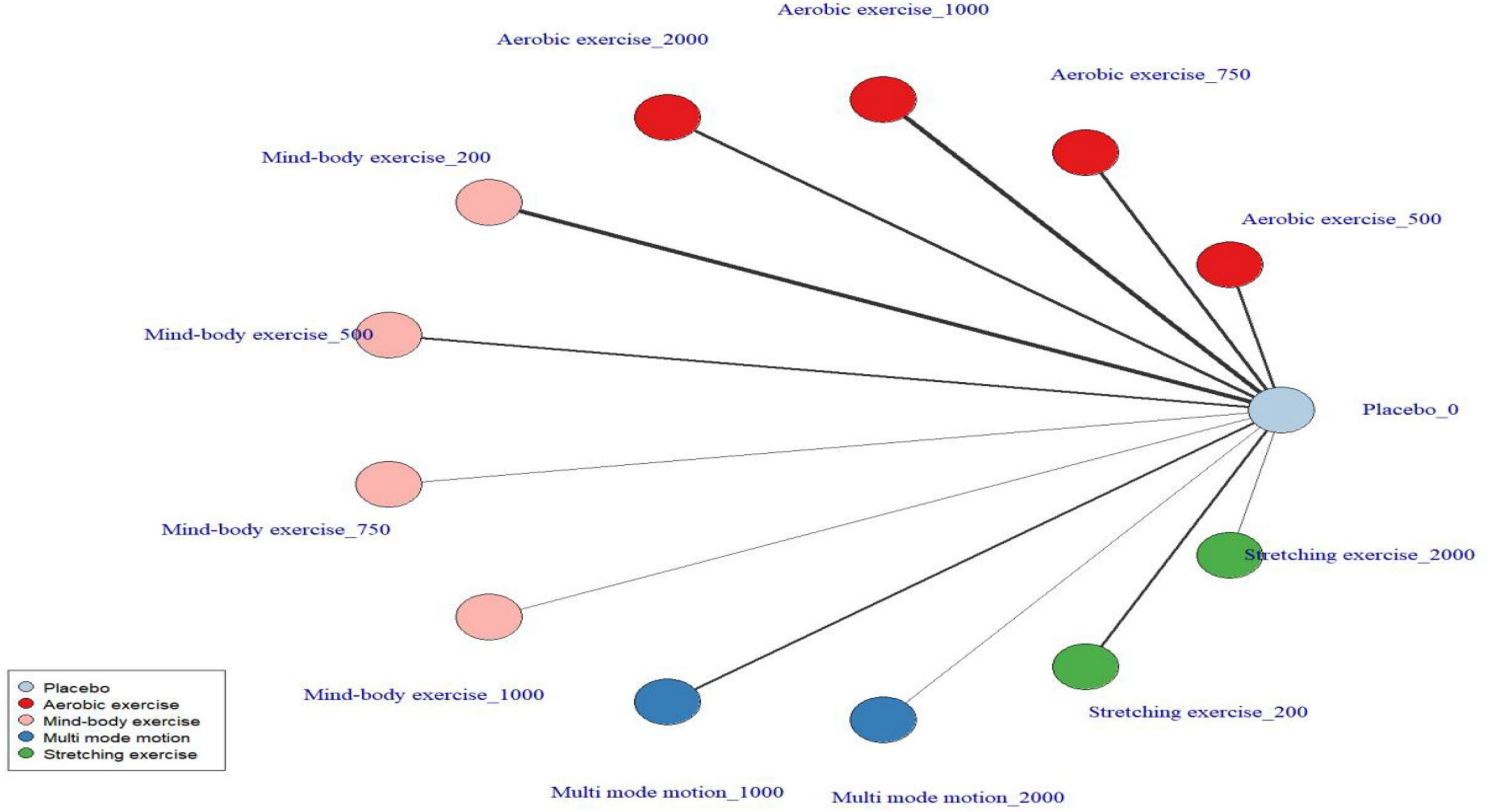
Exercise dose network diagram.

**Figure 5:**
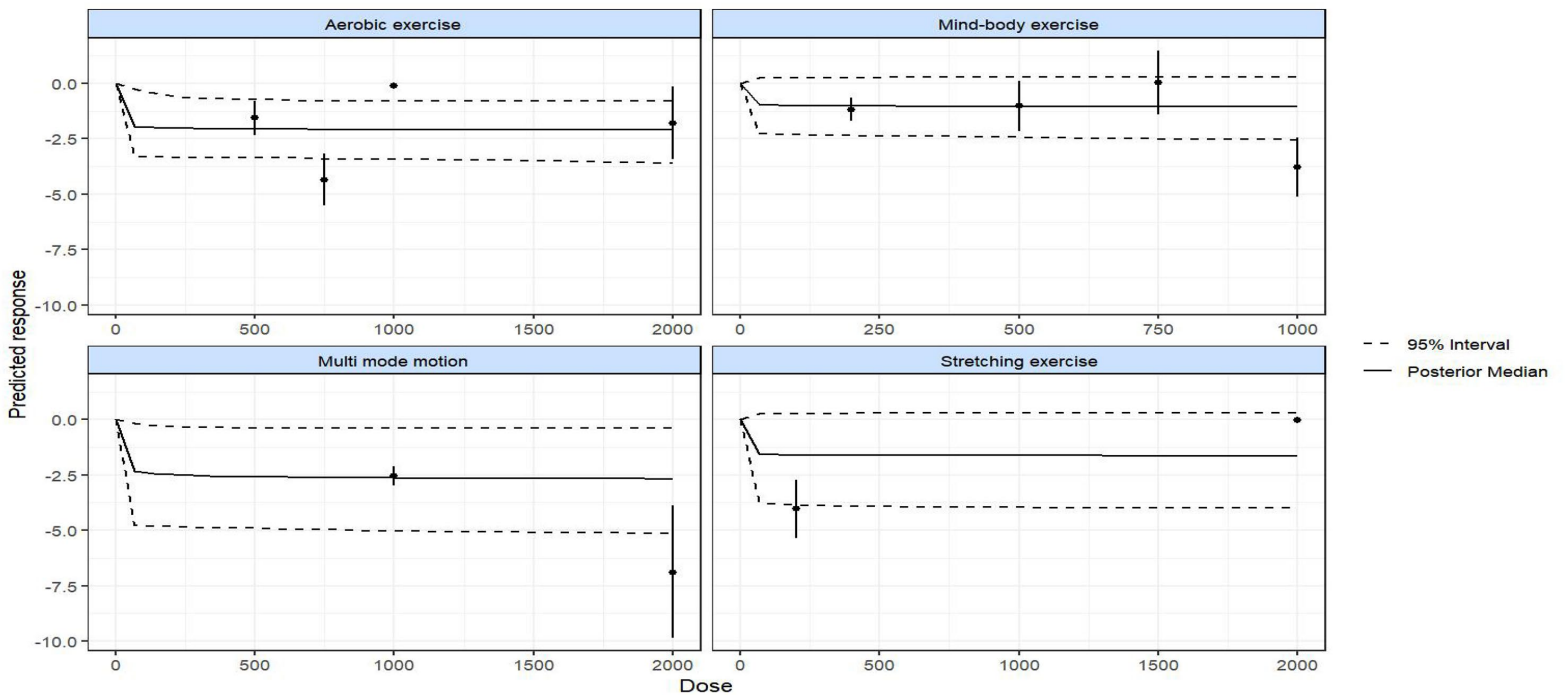
Dose–response relationships of different physical activity interventions.

**Figure 6:**
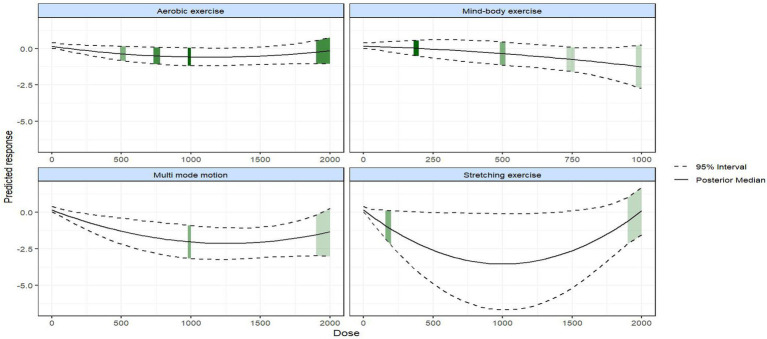
Dose–response curves of physical activity modalities on menopausal depression. The green shading indicates the sample size distribution across different exercise doses (the darker the color, the larger the sample size).

Prior to conducting modeling of exercise types and dose–response relationships, an evidence network was first constructed and evaluated (Figures 3, 4). This network includes six nodes: the placebo (no-intervention control, placebo_0) group and five dose groups corresponding to 200 METs, 500 METs, 750 METs, 1,000 METs, and 2000 METs per week, forming the foundational evidence framework for subsequent modeling (Figure 4). Predictions of the dose–response relationships for four types of exercise based on the Emax model (Figure 5, using “weekly MET-minutes” as the dose metric, and comparing the standardized effects on depression outcomes) indicate that: all exercise types fit the “saturation response hypothesis” of the Emax model—depressive symptom improvement increases rapidly with dose at low levels, while marginal benefits significantly decrease at higher doses. This establishes the overall effect characteristics for subsequent detailed analysis (Appendix C). Figure 6 further presents the dose–response relationships between aerobic, mind–body, multi-mode motion, and stretching exercises and menopausal depressive symptoms (the solid line represents the posterior median of effects, the dashed line represents the 95% CI, and the green shading represents the distribution of study numbers across different dose levels). The dose–response curves, based on the perspective of a second-order polynomial model (Appendix D), exhibit the following specific characteristics: multi-mode motion shows the most significant antidepressant benefits, with the efficacy peaking in the 1,000–1,500 MET-min/week range, and the 95% CI being narrow (indicating a high density of data in this dose range). No further efficacy improvement or even a reversal of effect is observed beyond this range; mind–body exercise exhibits a monotonically increasing effect with dose (without a saturation plateau), but at higher doses (>1,500 MET-min/week), the 95% CI widens (green shading lightens, indicating sparse data), leading to a high degree of uncertainty in effect estimates; stretching exercises follow a U-shaped curve, with the optimal efficacy occurring in the 900–1,200 MET-min/week range, while doses below 900 MET-min/week or above 1,200 MET-min/week result in diminished effects; aerobic exercise shows a mild saturation trend, with efficacy rising rapidly at lower to moderate doses (600–1,100 MET-min/week), and plateauing after 1,100 MET-min/week with a slight decline in effect (marginal diminishing returns). Quantified data for the complete dose gradient can be found in the Supplementary material.

**Figure 7:**
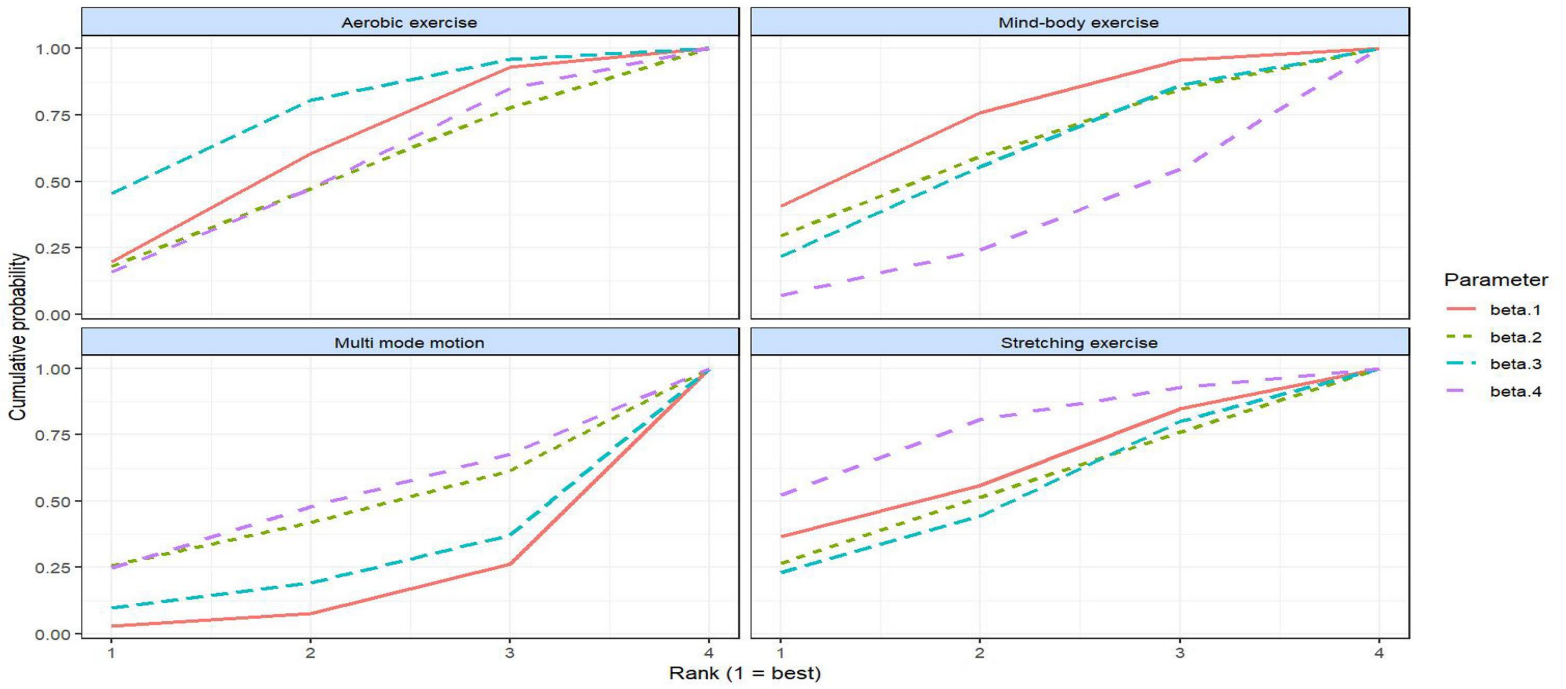
Cumulative rank probability plots and SUCRA scores for each exercise modality.

To quantitatively verify the dose–response nonlinearity pattern for the four types of exercise described above, node splitting analysis was first performed to confirm the consistency of the dose–response network (Appendix E). The results indicated that most contrast *p*-values were greater than 0.05, suggesting no significant conflicts between direct and indirect evidence, thereby indicating good network consistency. Subsequently, the posterior distributions of the natural spline coefficients (β₁–β₄) for each exercise type were analyzed using a non-parametric MBNMA model (Table 2). The core meanings of these four coefficients are as follows: β₁ (initial slope) reflects the direction of effects at the early stage of dosage increase, β₂ (curvature) represents the degree of non-linearity, β₃ (maximum efficacy) indicates the upper limit of the intervention’s optimal efficacy, and β₄ (tail/lag effect) describes the stability of effects at high doses or during long-term interventions. The posterior distribution results show that β₁ is predominantly negative (with more pronounced negative values for aerobic and mind–body exercises), confirming that depressive symptoms can consistently improve at initial doses. The distribution of β₂ aligns with the nonlinear characteristics of each exercise type: multi-mode motion has a β₂ close to 0 with low dispersion, corresponding to a stable plateau phase; stretching exercise has a bidirectional fluctuation in β₂, corresponding to a U-shaped curve; and aerobic exercise has a small positive β₂, indicating diminishing marginal benefits at high doses. The posterior median of β₃ is highest for mind–body exercise, suggesting a higher optimal efficacy limit, while the posterior median of β₄ is highest for stretching exercise, indicating greater stability of high-dose/long-term effects. No significant advantage was observed for multi-mode motion in either β₃ or β₄. Based on the aforementioned nonparametric MBNMA model, we further calculated the SUCRA values for each exercise type across the four parameters (β₁–β₄) and visualized the ranking probabilities using a cumulative probability plot (Figure 7). In the plot, higher curves indicate a greater probability of the exercise ranking better on the corresponding parameter. The SUCRA values range from 0 to 1, with higher values indicating better performance. The results show the following: for the β₁ (initial slope) dimension, mind–body exercise has the highest SUCRA value (0.151), followed by stretching exercise (0.131) and aerobic exercise (0.133), with multi-mode motion having the lowest (0.054); for the β₂ (curvature) dimension, mind–body exercise (0.130) > stretching exercise (0.119) > aerobic exercise (0.115) > multi-mode motion (0.104); for the β₃ (maximum efficacy) dimension, aerobic exercise (0.156) > stretching exercise (0.116) > mind–body exercise (0.127) > multi-mode motion (0.070); and for the β₄ (tail/lag effect) dimension, stretching exercise has the highest SUCRA value (0.156), significantly outperforming the other three exercise types. It should be noted that the current study’s data primarily focus on the moderate-to-low dose range (500–1,500 MET-min/week), with limited data available for high doses (>1,500 MET-min/week). This scarcity results in a reliable SUCRA value for β₄ (tail/delayed effects) only in stretching exercises, while estimates for β₄ in other types of exercise remain uncertain. As a result, the efficacy estimates at extreme doses are constrained. This conclusion is further supported by the fitting results of the basic UME model (Appendix F).

**Table 2:** SUCRA values for each model parameter and exercise modality.

Exercise modality	SUCRA_β₁	SUCRA_β₂	SUCRA_β₃	SUCRA_β₄
Aerobic exercise	0.133	0.115	**0.156**	0.119
Mind–body exercise	**0.151**	**0.13**	0.127	0.083
Multi-mode motion	0.054	0.104	0.07	0.111
Stretching exercise	0.131	0.119	0.116	**0.156**

Surface Under the Cumulative Ranking Curve (SUCRA) values derived from Bayesian model-based network meta-analysis (MBNMA) indicate the relative probability of each exercise modality being the most effective across four model parameters (β₁–β₄). Higher SUCRA scores suggest greater likelihood of ranking among the top-performing interventions. Each parameter captures a different aspect of the dose–response curve: β₁ (initial slope), β₂ (curvature), β₃ (maximum efficacy), and β₄ (tail or delayed effect). The bolded items indicate more significant effects.

**Figure 8:**
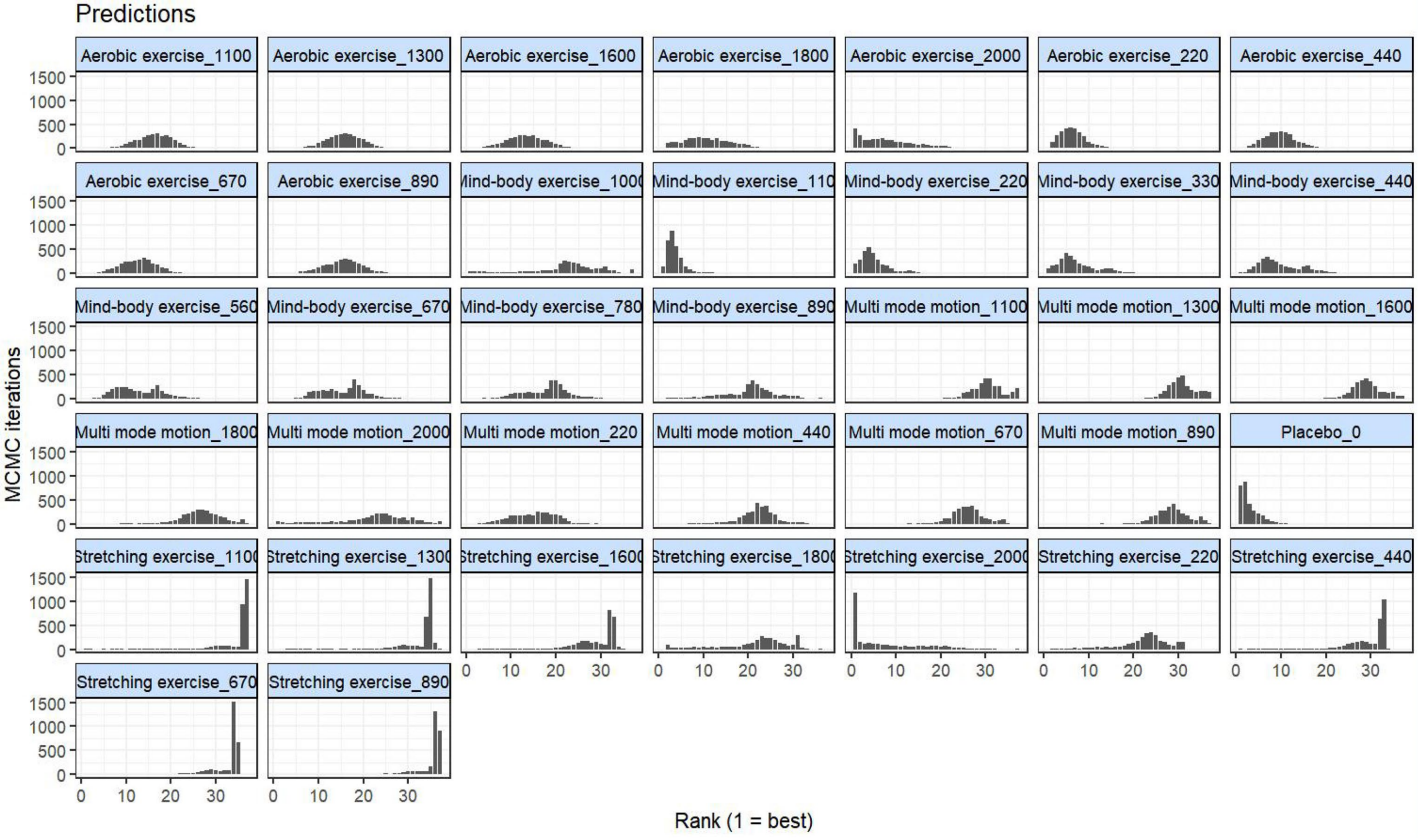
Posterior rank distributions of intervention–dose on menopausal depression. *X*-axis: Rank of intervention-dose combinations (1 = best); *Y*-axis: Rank prediction frequency, displaying relative efficacy probability of each exercise-dose combination and placebo.

Furthermore, the posterior rank distribution results of the network meta-analysis using the user-defined polynomial function (Figure 8) further support the above conclusions. The figure demonstrates that placebo consistently ranks the worst, while low-to-moderate doses of aerobic exercise (600–1,100 MET-min/week) and multi-mode motion (1000–1,500 MET-min/week) maintain higher rankings. In contrast, high-dose stretching exercise (>1,200 MET-min/week) ranks lower. This finding aligns with the previously stated dose–response characteristics: “aerobic exercise and multi-mode motion show superior efficacy at low-to-moderate doses, while the effect of high-dose stretching exercise diminishes.” These results further confirm the dose- and mode-dependent heterogeneity of exercise intervention efficacy, providing additional evidence for the recommendation of optimal dosages.

## 4 Discussion

This systematic review and network meta-analysis included 23 RCTs involving 2,155 climacteric women (1,202 in the intervention group, 953 in the control group; mean age > 45 years for both), evaluating the effects of aerobic exercise, mind–body exercises (e.g., yoga, Pilates, and qigong), stretching exercises, and multi-mode motion (combining aerobic, strength, or flexibility training) on their depressive symptoms. Overall, all exercise interventions significantly alleviated depressive symptoms in this group, with variations in effectiveness across types and a prominent dosage-efficacy relationship. Multi-mode motion showed peak antidepressant effects at 1,000–1,500 MET-min/week (narrow 95% CI, indicating stable effects) with no additional benefits beyond this range. Aerobic exercise efficacy rose rapidly around 600–1,100 MET-min/week, plateauing beyond 1,100 MET-min/week with slight marginal diminishing returns. Stretching exercise followed a U-shaped curve, with optimal effects only at 900–1,200 MET-min/week; both lower (<900 MET-min/week) and higher (>1,200 MET-min/week) doses reduced effectiveness. Mind–body exercise efficacy increased monotonically with dosage (no saturation plateau), but scarce data on high doses (>1,500 MET-min/week) led to considerable uncertainty in effect estimates.

Based on the results of the network meta-analysis, aerobic exercise and multi-mode motion were found to have a significantly higher probability of being the “best interventions” for alleviating depressive symptoms in climacteric women compared to mind–body exercise and stretching exercise, with the latter two showing comparable and generally favorable efficacy. Meanwhile, the analysis of the dose–response model confirmed that, with the exception of mind–body exercise, all other exercise types conformed to the saturation response hypothesis (Meyer et al., 2016). Specifically, in the low-dose phase, depressive symptoms improved rapidly as exercise dosage increased; however, upon reaching the high-dose phase, the marginal benefits of increasing the dose were significantly reduced (Watt, 2018). This finding suggests that moderate exercise is sufficient to achieve most of the improvement in depressive symptoms for climacteric women. Excessive exercise not only fails to further enhance efficacy, but may also place additional stress on the already vulnerable musculoskeletal system of climacteric women, potentially increasing physical discomfort that could impact emotional state.

From the perspective of exercise mechanisms, aerobic exercise relies on the rhythmic contraction of large muscle groups to activate the neuroendocrine system (Athanasiou et al., 2023; Dunn and Dishman, 1991). Clinically, it is suitable for women with acceptable joint function and a preference for a single exercise mode. The recommended prescription is 3 sessions per week, 40 min per session (e.g., brisk walking, cycling, intensity of approximately 6 METs), achieving a weekly dose of 720 MET-min/week, which falls within the optimal range. For those with joint discomfort, low-impact alternatives such as swimming can be chosen to avoid increasing physical burden while ensuring the antidepressant effect. Any further increase in dosage may induce fatigue, leading to a plateau in efficacy or even a slight decline.

Therefore, the optimal exercise dose is defined as 600–1,100 MET-min/week (Elavsky and McAuley, 2007; Pang and Kim, 2021; Bowen et al., 2006; Imayama et al., 2011; Bernard et al., 2015; Hu et al., 2017; Sternfeld et al., 2014; Abedi et al., 2015; Elsayed et al., 2022; Luoto et al., 2012; Noh et al., 2020; Sen et al., 2020). Multi-mode motion integrates aerobic training, strength training, and flexibility exercises, establishing a multi-mechanistic synergistic system that combines “aerobic regulation of hormone levels + strength to improve physical discomfort (such as back pain caused by muscle loss during menopause, which can exacerbate depressive mood) + stretching to relieve anxiety” (Vaughan et al., 2014; Baker et al., 2007). Due to the need to simultaneously activate multiple modules, this exercise type requires a higher dosage: when the weekly dose ranges between 1,000–1,500 MET-min/week, the synergistic effect reaches its peak. If the dosage exceeds this range, the cumulative load of the multiple modules may exceed the body’s tolerance limit, which could compromise the antidepressant benefits due to potential physical strain (Ganjeh et al., 2025).

Stretching exercise, primarily focusing on static or dynamic muscle stretching, exerts antidepressant effects by alleviating physical tension (Aibar-Almazán et al., 2019; Afonso et al., 2012; Payne and Crane-Godreau, 2013). For climacteric women, fluctuations in hormone levels often lead to muscle stiffness and joint discomfort, which can further intensify depressive symptoms (Wang et al., 2025). The dose-effect relationship for stretching exercise exhibits a “U-shaped” pattern, mainly due to the existence of bidirectional load thresholds: when the weekly dose is below 900 MET-min/week, the intensity and frequency of exercise are insufficient to effectively relax muscles or alleviate joint stiffness, making it difficult to improve somatic symptom-related depression. When the weekly dose exceeds 1,200 MET-min/week, it is important to note the limitations of the available evidence: as shown in Figure 6, the data density in this range (indicated by green shading) is sparse, and the 95% credible interval widens significantly. The observed downward trend in efficacy from the quadratic function model may be a mathematical artifact rather than confirmed clinical deterioration. Therefore, there is insufficient evidence to draw a definitive conclusion about the effect of high-dose stretching exercise—we cannot assert it is harmful or exacerbates depressive symptoms, only that the current data do not support clear efficacy beyond the optimal range of 900–1,200 MET-min/week.

Mind–body exercises (e.g., yoga and Pilates) adopt an integrated “physical activity + psychological regulation” model (Elavsky and McAuley, 2007; Aibar-Almazán et al., 2019; Abdoshahi, 2023; Afonso et al., 2012; Li et al., 2022; Newton et al., 2014; del Carmen Carcelén-Fraile et al., 2022; Arslan Kabasakal, 2025). Their mechanisms include activating the parasympathetic nervous system via breathing exercises to alleviate sympathetic overactivity-related anxiety, and reducing negative emotional cognitive processing through meditation. With low physiological load and regulatory mechanisms that rarely reach saturation (Li et al., 2020; Dong et al., 2024), their therapeutic effects appeared to increase monotonically with dosage (e.g., higher frequency or longer meditation duration). However, the upper limit of efficacy remains unclear due to scarce high-dose intervention data. Notably, climacteric women’s abrupt estrogen decline impairs regulatory capacity (Platt et al., 2025; Monteleone et al., 2018), significantly reducing tolerance to high-dose aerobic exercise (>1,100 MET-min/week) and potentially triggering hormonal fluctuations (e.g., elevated cortisol) that may exacerbate depressive symptoms (Mandrup et al., 2017).

In contrast, mind–body exercises require less hormonal regulation and are less influenced by physiological changes, resulting in a broader dose tolerance range (Payne and Crane-Godreau, 2013; Khadilkar, 2019). The physiological changes in the musculoskeletal system (including muscle loss, decreased bone density, and potential joint cartilage changes) create differential constraints on the dose tolerance of various types of exercise. Among them, stretching exercises have the highest dose sensitivity, directly affecting muscles and joints, and their optimal dose range is the narrowest (spanning only 300 MET-min/week); multi-mode motion, integrating strength training, must balance the “muscle stimulation effect” with “potential strain risk”; although aerobic exercise exerts less joint load compared to the other two types, high doses still may significantly increase the cardiovascular and joint burden (Athanasiou et al., 2023; Dunn and Dishman, 1991), with the optimal dose range for the latter two types spanning 500 MET-min/week. Mind–body exercises place the least load on the musculoskeletal system and have the most lenient dose limits (Li et al., 2020; Dong et al., 2024; Wise et al., 1999).

Regarding exercise prescription parameter matching, the included studies’ median exercise frequency (3 sessions/week) naturally aligns with the optimal doses of various exercises. Specifically: aerobic exercise (3 sessions/week, 40 min/session, ~6 METs) yields 720 MET-min/week, within the 600–1,100 MET-min/week optimal range; multi-mode motion (3 sessions/week, 50 min/session, 30 min aerobic + 20 min strength training, ~8–10 METs combined) achieves ~1,200–1,500 MET-min/week, highly consistent with its 1,000–1,500 MET-min/week optimal range; stretching exercise (3 sessions/week, 40 min/session, ~7–8 METs) results in ~840–960 MET-min/week (near the 900–1,200 MET-min/week optimal range’s lower limit), and extending sessions to 50 min meets optimal dose needs. For intervention duration, the median was 12 weeks—aerobic interventions of 8 weeks to 12 months consistently improved depression scores. This cycle balances treatment stability and adherence by avoiding short-term (<8 weeks) ineffectiveness from insufficient dose accumulation and long-term (>24 weeks) reduced adherence due to high-dose-related fatigue (Khalafi et al., 2023; Dishman, 1986).

This study has certain limitations. The scarcity of data for high doses (>1,500 MET-min/week) limits a comprehensive understanding of the dose-efficacy relationship for mind–body exercise. Furthermore, individual differences such as baseline estrogen levels and physical activity history were not further analyzed in terms of their impact on dosing, which may influence the “individualized adaptability” of the optimal dosage. However, from a clinical practice perspective, these dosage variations provide precise guidance: clinicians can formulate personalized exercise plans based on the patient’s physical condition, exercise preferences, and baseline assessments (e.g., depression severity, musculoskeletal health). For severe cases, a combination of multi-mode motion and psychological interventions can be considered. Future research should address the lack of data in the high-dose range and further refine the dose-efficacy relationship, while also incorporating the collection of adverse event data to verify potential physical strain risks associated with high-dose exercise, facilitating a shift in menopausal depression exercise interventions from “unified recommendations” to “individualized adaptation.”

### 4.1 Strengths and limitations

The strengths of this study lie in its rigorous methodology, adherence to the PRISMA 2020 guidelines, and registration with PROSPERO (CRD 420251162965). Following the PICOS framework, only randomized controlled trials (RCTs) were included. The reliability was ensured through independent screening by two researchers (*κ* = 0.71 for titles/abstracts, *κ* = 0.84 for full texts). A total of 23 RCTs involving 2,155 climacteric women were included, with depression assessed using validated tools such as the GDS and the HADS. The study employed MBNMA to clarify the efficacy and optimal dosages of four types of exercise, with rankings quantified using SUCRA values. This study fills the research gap of “emphasizing exercise type over dosage,” while also addressing the dose-efficacy differences in line with the physiological characteristics of climacteric women, providing biologically plausible conclusions.

However, this study has certain limitations. First, the included studies exhibit significant heterogeneity in intervention duration (ranging from 3 weeks to 12 months). Short- and long-term interventions differ markedly in cumulative dose, but our dose–response model focuses solely on weekly flux (MET-min/week) rather than total cumulative exposure over the entire intervention period. Consequently, it fails to distinguish the effects of identical weekly doses administered across different durations, potentially biasing the estimated optimal doses—given that varying physiological responses (e.g., neuroendocrine adaptation, musculoskeletal tolerance) are not accounted for. Other limitations include a risk of bias in some included studies, the inclusion of only English-language literature, insufficient analysis of how baseline characteristics influence optimal dosage, wide 95% confidence intervals for certain exercise comparisons, and potential publication bias (identified via Egger’s test). Despite statistical adjustments, the results therefore necessitate cautious interpretation.

### 4.2 Practical implications

These findings provide actionable guidance for clinical practice, community health, and individual management to alleviate depressive symptoms in climacteric women. For clinicians, the optimal dose of aerobic exercise is 600–1,100 MET-min/week, recommended at 3 sessions per week, each lasting 40 min (e.g., brisk walking, cycling). For those with joint discomfort, low-impact alternatives (e.g., swimming) may be chosen. The optimal dose for multi-mode motion is 1,000–1,500 MET-min/week, suitable for individuals with good joint function or a preference for varied exercise types, with 3 sessions per week, each lasting 50 min (30 min of aerobic exercise + 20 min of strength training). The optimal dose for stretching exercises is 900–1,200 MET-min/week, suitable for individuals with musculoskeletal pain or mobility issues, with 3 sessions per week, each lasting 40–50 min. Mind–body exercise can complement care for those with pronounced anxiety or hormone sensitivity; however, its short-term effects are less effective than aerobic exercise. The recommended dose is 1,000–1,500 MET-min/week, with 3–4 sessions per week, each lasting 45 min (e.g., yoga).

For communities: Prioritize group-based aerobic or multi-mode motion courses adhering to curve vertex-aligned doses (aerobic 600–1,100 MET-min/week, multi-mode 1,000–1,500 MET-min/week). Offer flexible scheduling and childcare to reduce barriers, diverse formats (e.g., dance-based aerobics), and “exercise prescriptions” linking exercise to medical care. Educate on “moderate dosage”—notably, 3 × 40 min/week aerobic exercise (curve-aligned peak efficacy) is effective—to dispel myths that exercise worsens menopausal discomfort. For individuals: Start with 3 × 15–20 min/week low-intensity aerobic exercise (e.g., post-meal walking), gradually advancing to curve vertex-aligned doses. Integrate exercise into daily routines (e.g., commute walking) and monitor weekly MET-min values to avoid under- or over-dosage. Support groups enhance adherence. Severe cases or those with comorbidities should begin with supervised multi-mode or mind–body exercise. In resource-limited settings, low-cost options (e.g., 3 × 30 min/week community walks; 3 × 30 × 7 METs = 630 MET-min/week, within aerobic’s optimal range) are viable.

In conclusion, clinicians should deliver personalized exercise plans based on baseline assessments, with doses mapped to respective dose–response curve vertices. Communities should provide accessible standard-dose courses, and individuals should develop sustainable habits. This approach maximizes the antidepressant benefits of aerobic, multi-mode, and stretching exercises, improves climacteric women’s mental health, reduces medication dependence, and empowers them during the menopausal transition.

## 5 Conclusion

This systematic review and network meta-analysis evaluated the effects of four types of exercise on depressive symptoms. The four structured exercise modalities—aerobic exercise, multi-mode motion, stretching exercise, and mind–body exercise—all significantly alleviate depressive symptoms in climacteric women. However, there are differences in the optimal dosage for each modality: aerobic exercise is most effective at a dose of 600–1,100 MET-min/week (with a plateau effect beyond 1,100 MET-min/week); multi-mode motion reaches peak efficacy at 1000–1500 MET-min/week (with excessive doses potentially increasing physical strain); stretching exercise follows a “U-shaped” curve, with optimal effects at 900–1200 MET-min/week; and mind–body exercise shows a dose-dependent improvement, although data on high doses (>1,500 MET-min/week) are scarce. The median exercise frequency of 3 sessions per week and the median intervention duration of 12 weeks naturally align with the optimal doses for all exercise types. These findings provide evidence for precision exercise interventions in menopausal depression and offer guidance for shifting intervention strategies from “unified recommendations” to “individualized adaptation.”

## Data Availability

The original contributions presented in the study are included in the article/Supplementary material, further inquiries can be directed to the corresponding authors.
