# Supplementary material for: Optimal doses of different exercise types for improving depressive symptoms in climacteric women: a systematic review and network meta-analysis
Source: Front Psychol. 2026 Apr 7;17:1727315. doi: 10.3389/fpsyg.2026.1727315 (PMC13096043; doi:10.3389/fpsyg.2026.1727315)

**Optimal Doses of Different Exercise Types for Improving Depressive**

**Symptoms in Climacteric Women: A Systematic Review and**

**Network Meta-Analysis**

Contents

[Appendix A PRISMA checklist 3](#_Toc1461093983)

[Appendix A1 PRISMA 2020 checklist 3](#_Toc131068836)

[Appendix A2 PRISMA NMA Checklist 6](#_Toc1703188477)

[Appendix B Search strategy 10](#_Toc1679202076)

[Appendix B1 Initial search strategy 10](#_Toc119202458)

[Appendix C Dose-response effects from NMA 16](#_Toc1980952602)

[Appendix D Quadratic dose–response curves for physical activity interventions estimated from a user-defined polynomial MBNMA model with 95% credible intervals. 17](#_Toc1431402373)

[Appendix E Node-Splitting Analysis of Inconsistency in Dose-Response Network 18](#_Toc1467869317)

[Appendix F Full UME Model Results 20](#_Toc187474083)

[Table S1 Dose–Response Effects of Exercise Modalities on Menopausal Depression 21](#_Toc518402832)

[Table S2 Results of Network Meta-Analysis 22](#_Toc455241545)

[Figure S1 Posterior Distributions of Spline Parameters (β₁, β₂) by Exercise Type in Nonparametric MBNMA 24](#_Toc1907896201)

[Figure S2 NMA-estimated dose–response curve for physical activity interventions. 25](#_Toc1933116850)

[FIgure S3 RCS-Estimated Dose–Response Curves for Exercise Interventions: Solid Lines Denote Posterior Medians; Dashed Lines 95% Credible Intervals 26](#_Toc614802487)

# **Appendix A** PRISMA checklist

## **Appendix A1** PRISMA 2020 checklist

| **Section and Topic** | **Item #** | **Checklist item** | **Location where item is reported** |
| --- | --- | --- | --- |
| **TITLE** | | |  |
| Title | 1 | Identify the report as a systematic review. | Page 1 |
| **ABSTRACT** | | |  |
| Abstract | 2 | See the PRISMA 2020 for Abstracts checklist. | Page 2-3 |
| **INTRODUCTION** | | |  |
| Rationale | 3 | Describe the rationale for the review in the context of existing knowledge. | Page 4-7 |
| Objectives | 4 | Provide an explicit statement of the objective(s) or question(s) the review addresses. | Page 4-7 |
| **METHODS** | | |  |
| Eligibility criteria | 5 | Specify the inclusion and exclusion criteria for the review and how studies were grouped for the syntheses. | Page7-10 |
| Information sources | 6 | Specify all databases, registers, websites, organisations, reference lists and other sources searched or consulted to identify studies. Specify the date when each source was last searched or consulted. | Page 7-10 |
| Search strategy | 7 | Present the full search strategies for all databases, registers and websites, including any filters and limits used. | Page 8-10 |
| Selection process | 8 | Specify the methods used to decide whether a study met the inclusion criteria of the review, including how many reviewers screened each record and each report retrieved, whether they worked independently, and if applicable, details of automation tools used in the process. | Page 9-10 |
| Data collection process | 9 | Specify the methods used to collect data from reports, including how many reviewers collected data from each report, whether they worked independently, any processes for obtaining or confirming data from study investigators, and if applicable, details of automation tools used in the process. | Page10-11 |
| Data items | 10a | List and define all outcomes for which data were sought. Specify whether all results that were compatible with each outcome domain in each study were sought (e.g. for all measures, time points, analyses), and if not, the methods used to decide which results to collect. | Page 10-11 |
|  | 10b | List and define all other variables for which data were sought (e.g. participant and intervention characteristics, funding sources). Describe any assumptions made about any missing or unclear information. | Page 9-11 |
| Study risk of bias assessment | 11 | Specify the methods used to assess risk of bias in the included studies, including details of the tool(s) used, how many reviewers assessed each study and whether they worked independently, and if applicable, details of automation tools used in the process. | Page 12 |
| Effect measures | 12 | Specify for each outcome the effect measure(s) (e.g. risk ratio, mean difference) used in the synthesis or presentation of results. | Page 12-13 |
| Synthesis methods | 13a | Describe the processes used to decide which studies were eligible for each synthesis (e.g. tabulating the study intervention characteristics and comparing against the planned groups for each synthesis (item #5)). | Page 12-13 |
|  | 13b | Describe any methods required to prepare the data for presentation or synthesis, such as handling of missing summary statistics, or data conversions. | Page 12-13 |
|  | 13c | Describe any methods used to tabulate or visually display results of individual studies and syntheses. | Page 12-13 |
|  | 13d | Describe any methods used to synthesize results and provide a rationale for the choice(s). If meta-analysis was performed, describe the model(s), method(s) to identify the presence and extent of statistical heterogeneity, and software package(s) used. | Page 12-13 |
|  | 13e | Describe any methods used to explore possible causes of heterogeneity among study results (e.g. subgroup analysis, meta-regression). | Page 12-13 |
|  | 13f | Describe any sensitivity analyses conducted to assess robustness of the synthesized results. | Page 12-13 |
| Reporting bias assessment | 14 | Describe any methods used to assess risk of bias due to missing results in a synthesis (arising from reporting biases). | Page 11-12 |
| Certainty assessment | 15 | Describe any methods used to assess certainty (or confidence) in the body of evidence for an outcome. | Page 11-12 |
| **RESULTS** | | |  |
| Study selection | 16a | Describe the results of the search and selection process, from the number of records identified in the search to the number of studies included in the review, ideally using a flow diagram. | Page 13-14 |
|  | 16b | Cite studies that might appear to meet the inclusion criteria, but which were excluded, and explain why they were excluded. | Page 13-15 |
| Study characteristics | 17 | Cite each included study and present its characteristics. | Page 14-15 |
| Risk of bias in studies | 18 | Present assessments of risk of bias for each included study. | Page 14 |
| Results of individual studies | 19 | For all outcomes, present, for each study: (a) summary statistics for each group (where appropriate) and (b) an effect estimate and its precision (e.g. confidence/credible interval), ideally using structured tables or plots. | Page 14-15 |
| Results of syntheses | 20a | For each synthesis, briefly summarise the characteristics and risk of bias among contributing studies. | Page 15-16 |
|  | 20b | Present results of all statistical syntheses conducted. If meta-analysis was done, present for each the summary estimate and its precision (e.g. confidence/credible interval) and measures of statistical heterogeneity. If comparing groups, describe the direction of the effect. | Page 16-19 |
|  | 20c | Present results of all investigations of possible causes of heterogeneity among study results. | Page 16-19 |
|  | 20d | Present results of all sensitivity analyses conducted to assess the robustness of the synthesized results. | Page 16-19 |
| Reporting biases | 21 | Present assessments of risk of bias due to missing results (arising from reporting biases) for each synthesis assessed. | Page 15-19 |
| Certainty of evidence | 22 | Present assessments of certainty (or confidence) in the body of evidence for each outcome assessed. | Page 16-19 |
| **DISCUSSION** | | |  |
| Discussion | 23a | Provide a general interpretation of the results in the context of other evidence. | Page 20-26 |
|  | 23b | Discuss any limitations of the evidence included in the review. | Page 25-26 |
|  | 23c | Discuss any limitations of the review processes used. | Page 27-28 |
|  | 23d | Discuss implications of the results for practice, policy, and future research. | Page 28-30 |
| **OTHER INFORMATION** | | |  |
| Registration and protocol | 24a | Provide registration information for the review, including register name and registration number, or state that the review was not registered. | Page 7-8 |
|  | 24b | Indicate where the review protocol can be accessed, or state that a protocol was not prepared. | Page 7-8 |
|  | 24c | Describe and explain any amendments to information provided at registration or in the protocol. | Page 7-8 |
| Support | 25 | Describe sources of financial or non-financial support for the review, and the role of the funders or sponsors in the review. | Page 30-31 |
| Competing interests | 26 | Declare any competing interests of review authors. | Page 30-31 |
| Availability of data, code and other materials | 27 | Report which of the following are publicly available and where they can be found: template data collection forms; data extracted from included studies; data used for all analyses; analytic code; any other materials used in the review. | Page 30-31 |

## **Appendix A2** PRISMA NMA Checklist

| **Section/Topic** | **Item #** | **Checklist Item** | **Reported on Page #** |
| --- | --- | --- | --- |
| **TITLE** |  |  |  |
| Title | 1 | Identify the report as a systematic review *incorporating a network meta-analysis (or related form of meta-analysis).* | 1 |
|  |  |  |  |
| **ABSTRACT** |  |  |  |
| Structured summary | 2 | Provide a structured summary including, as applicable:  **Background:** main objectives  **Methods:** data sources; study eligibility criteria, participants, and interventions; study appraisal; and *synthesis methods, such as network meta-analysis.*  **Results:** number of studies and participants identified; summary estimates with corresponding confidence/credible intervals; *treatment rankings may also be discussed. Authors may choose to summarize pairwise comparisons against a chosen treatment included in their analyses for brevity.*  **Discussion/Conclusions:** limitations; conclusions and implications of findings.  **Other:** primary source of funding; systematic review registration number with registry name. | 2-3 |
|  |  |  |  |
| **INTRODUCTION** |  |  |  |
| Rationale | 3 | Describe the rationale for the review in the context of what is already known*, including mention of why a network meta-analysis has been conducted.* | 4-6 |
| Objectives | 4 | Provide an explicit statement of questions being addressed, with reference to participants, interventions, comparisons, outcomes, and study design (PICOS). | 6-7 |
|  |  |  |  |
| **METHODS** |  |  |  |
| Protocol and registration | 5 | Indicate whether a review protocol exists and if and where it can be accessed (e.g., Web address); and, if available, provide registration information, including registration number. | 7 |
| Eligibility criteria | 6 | Specify study characteristics (e.g., PICOS, length of follow-up) and report characteristics (e.g., years considered, language, publication status) used as criteria for eligibility, giving rationale. *Clearly describe eligible treatments included in the treatment network, and note whether any have been clustered or merged into the same node (with justification).* | 7-12 |
| Information sources | 7 | Describe all information sources (e.g., databases with dates of coverage, contact with study authors to identify additional studies) in the search and date last searched. | 7-8 |
| Search | 8 | Present full electronic search strategy for at least one database, including any limits used, such that it could be repeated. | 7-8 |
| Study selection | 9 | State the process for selecting studies (i.e., screening, eligibility, included in systematic review, and, if applicable, included in the meta-analysis). | 8-10 |
| Data collection process | 10 | Describe method of data extraction from reports (e.g., piloted forms, independently, in duplicate) and any processes for obtaining and confirming data from investigators. | 10-12 |
| Data items | 11 | List and define all variables for which data were sought (e.g., PICOS, funding sources) and any assumptions and simplifications made. | 10-12 |
| **Geometry of the network** | **S1** | Describe methods used to explore the geometry of the treatment network under study and potential biases related to it. This should include how the evidence base has been graphically summarized for presentation, and what characteristics were compiled and used to describe the evidence base to readers. | 12-13 |
| Risk of bias within individual studies | 12 | Describe methods used for assessing risk of bias of individual studies (including specification of whether this was done at the study or outcome level), and how this information is to be used in any data synthesis. | 12 |
| Summary measures | 13 | State the principal summary measures (e.g., risk ratio, difference in means). *Also describe the use of additional summary measures assessed, such as treatment rankings and surface under the cumulative ranking curve (SUCRA) values, as well as modified approaches used to present summary findings from meta-analyses.* | 12-13 |
| Planned methods of analysis | 14 | Describe the methods of handling data and combining results of studies for each network meta-analysis. This should include, but not be limited to:   - *Handling of multi-arm trials;* - *Selection of variance structure;* - *Selection of prior distributions in Bayesian analyses; and* - *Assessment of model fit.* | 12-13 |
| **Assessment of Inconsistency** | **S2** | Describe the statistical methods used to evaluate the agreement of direct and indirect evidence in the treatment network(s) studied. Describe efforts taken to address its presence when found. | 12-13 |
| Risk of bias across studies | 15 | Specify any assessment of risk of bias that may affect the cumulative evidence (e.g., publication bias, selective reporting within studies). | 12-13 |
| Additional analyses | 16 | Describe methods of additional analyses if done, indicating which were pre-specified. This may include, but not be limited to, the following:   - Sensitivity or subgroup analyses; - Meta-regression analyses; - *Alternative formulations of the treatment network; and* - *Use of alternative prior distributions for Bayesian analyses (if applicable).* | 12-13 |
|  |  |  |  |
| **RESULTS** |  |  |  |
| Study selection | 17 | Give numbers of studies screened, assessed for eligibility, and included in the review, with reasons for exclusions at each stage, ideally with a flow diagram. | 13-14 |
| **Presentation of network structure** | **S3** | Provide a network graph of the included studies to enable visualization of the geometry of the treatment network. | 14-16 |
| **Summary of network geometry** | **S4** | Provide a brief overview of characteristics of the treatment network. This may include commentary on the abundance of trials and randomized patients for the different interventions and pairwise comparisons in the network, gaps of evidence in the treatment network, and potential biases reflected by the network structure. | 15-18 |
| Study characteristics | 18 | For each study, present characteristics for which data were extracted (e.g., study size, PICOS, follow-up period) and provide the citations. | 14-18 |
| Risk of bias within studies | 19 | Present data on risk of bias of each study and, if available, any outcome level assessment. | 15-16 |
| Results of individual studies | 20 | For all outcomes considered (benefits or harms), present, for each study: 1) simple summary data for each intervention group, and 2) effect estimates and confidence intervals. *Modified approaches may be needed to deal with information from larger networks.* | 16-18 |
| Synthesis of results | 21 | Present results of each meta-analysis done, including confidence/credible intervals. *In larger networks, authors may focus on comparisons versus a particular comparator (e.g. placebo or standard care), with full findings presented in an appendix. League tables and forest plots may be considered to summarize pairwise comparisons.* If additional summary measures were explored (such as treatment rankings), these should also be presented. | 14-19 |
| **Exploration for inconsistency** | **S5** | Describe results from investigations of inconsistency. This may include such information as measures of model fit to compare consistency and inconsistency models, *P* values from statistical tests, or summary of inconsistency estimates from different parts of the treatment network. | 16-19 |
| Risk of bias across studies | 22 | Present results of any assessment of risk of bias across studies for the evidence base being studied. | 16-19 |
| Results of additional analyses | 23 | Give results of additional analyses, if done (e.g., sensitivity or subgroup analyses, meta-regression analyses*, alternative network geometries studied, alternative choice of prior distributions for Bayesian analyses,* and so forth). | 16-19 |
|  |  |  |  |
| **DISCUSSION** |  |  |  |
| Summary of evidence | 24 | Summarize the main findings, including the strength of evidence for each main outcome; consider their relevance to key groups (e.g., healthcare providers, users, and policy-makers). | 19-25 |
| Limitations | 25 | Discuss limitations at study and outcome level (e.g., risk of bias), and at review level (e.g., incomplete retrieval of identified research, reporting bias). *Comment on the validity of the assumptions, such as transitivity and consistency. Comment on any concerns regarding network geometry (e.g., avoidance of certain comparisons).* | 26-27 |
| Conclusions | 26 | Provide a general interpretation of the results in the context of other evidence, and implications for future research. | 29-30 |
|  |  |  |  |
| **FUNDING** |  |  |  |
| Funding | 27 | Describe sources of funding for the systematic review and other support (e.g., supply of data); role of funders for the systematic review. This should also include information regarding whether funding has been received from manufacturers of treatments in the network and/or whether some of the authors are content experts with professional conflicts of interest that could affect use of treatments in the network. | 31 |

# **Appendix B** Search strategy

## **Appendix B1** Initial search strategy

| **Database** | **Search strategy** | **amount** |
| --- | --- | --- |
| **PubMed** | | |
| #1 | **(((((((((Depressive Symptoms[MeSH Terms]) OR ([Depressive Symptoms[Title/Abstract])) OR (Depressive Symptom[Title/Abstract])) OR (Emotional Depression[Title/Abstract])) OR (Symptom, Depressive[Title/Abstract])) OR (Depression, Emotional[Title/Abstract])) OR (Negative Emotion[Title/Abstract])) OR (Negative Mood[Title/Abstract])) OR (Affective Disorder[Title/Abstract])) OR (Affective Disorders[Title/Abstract]))** | 240,103   results |
| #2 | **(((((((((((climacteric women[MeSH Terms])) OR (climacteric women[Title/Abstract]) OR (menopausal woman[Title/Abstract])) OR (menopausal women[Title/Abstract])) OR (menopause females[Title/Abstract])) OR (menopause women[Title/Abstract])) OR (menopause feminine[Title/Abstract])) OR (menopause female[Title/Abstract])) OR (menopausal[Title/Abstract])) OR (postmenopause[Title/Abstract])) OR (postmenopausal[Title/Abstract])) OR (climacteric female[Title/Abstract]))** | 96,168  results |
| #3 | Search:**((((((((((((((((((((((((((Exercise[MeSH Terms]) OR (Exercises[Title/Abstract])) OR (Physical Activity[Title/Abstract])) OR (Activities, Physical[Title/Abstract])) OR (Activity, Physical[Title/Abstract])) OR (Physical Activities[Title/Abstract])) OR (Exercise, Physical[Title/Abstract])) OR (Exercises, Physical[Title/Abstract])) OR (Physical Exercise[Title/Abstract])) OR (Physical Exercises[Title/Abstract])) OR (Acute Exercise[Title/Abstract])) OR (Acute Exercises[Title/Abstract])) OR (Exercise, Acute[Title/Abstract])) OR (Exercises, Acute[Title/Abstract])) OR (Exercise, Isometric[Title/Abstract])) OR (Exercises, Isometric[Title/Abstract])) OR (Isometric Exercises[Title/Abstract])) OR (Isometric Exercise[Title/Abstract])) OR (Exercise, Aerobic[Title/Abstract])) OR (Aerobic Exercise[Title/Abstract])) OR (Aerobic Exercises[Title/Abstract])) OR (Exercises, Aerobic[Title/Abstract])) OR (Exercise Training[Title/Abstract])) OR (Exercise Trainings[Title/Abstract])) OR (Training, Exercise[Title/Abstract])) OR (Trainings, Exercise[Title/Abstract])) OR (((((((((((((((Aerobic[Title/Abstract]) OR (Jog[Title/Abstract])) OR (Walk[Title/Abstract])) OR (Pilates[Title/Abstract])) OR (Strength training[Title/Abstract])) OR (Stretching[Title/Abstract])) OR (Ambulation[Title/Abstract])) OR (Yoga[Title/Abstract])) OR (Swim[Title/Abstract])) OR (Dance [Title/Abstract])) OR (Dancing[Title/Abstract])) OR (strengthening[Title/Abstract])) OR (warming up[Title/Abstract])) OR (cooling down[Title/Abstract])))** | 738,888 results |
| #4 | #1 AND #2 AND#3 | 204 results |

| **Embase** | | |
| --- | --- | --- |
| #1 | **'Depression'/exp OR 'Depression' OR '**Depressive Symptom**' OR '**Depressive Symptoms**' OR '**Emotional Depression**' OR** 'Symptom, Depressive' **OR** 'Depression, Emotional**' OR '**Negative Emotion**' OR '**Negative Mood**' OR '**Affective Disorder**' OR '**Affective Disorders**'** | 1,159,951 |
| #2 | '**climacteric women**'/exp OR '**climacteric women**' OR '**menopausal woman**' OR '**menopausal women**' OR '**menopause females**' OR '**menopause women**' OR '**climacteric**' OR '**menopause feminine**' OR '**menopause female**' OR '**menopausal**' OR '**postmenopause**' OR '**postmenopausal**' OR '**climacteric female**' | 186,509 |
| #3 | **'Exercise'**/exp OR **'Exercise'** OR **'Exercises'** OR **'Physical Activity'** OR **'Activities, Physical'** OR **'Activity, Physical'** OR **'Physical Activities'** OR **'Exercise, Physical' OR 'Exercises, Physical' OR 'Physical Exercise' OR 'Physical Exercises' OR 'Acute Exercise' OR 'Acute Exercises' OR 'Exercise, Acute' OR 'Exercises, Acute' OR 'Exercise, Isometric' OR 'Exercises, Isometric' OR 'Isometric Exercises'** OR '**Isometric Exercise**' OR '**Exercise, Aerobic**' OR '**Aerobic Exercise**' OR '**Aerobic Exercises**' OR '**Exercises, Aerobic**' OR '**Exercise Training**' OR '**Exercise Trainings**' OR '**Training, Exercise**' OR '**Trainings, Exercise**' OR '**Aerobic**' OR '**Jog**' OR '**Walk**' OR '**Pilates**' OR '**Strength training**' OR '**Stretching**' OR '**Ambulation**' OR '**Yoga**' OR '**Swim**' OR '**Dance**' OR '**Dancing**' OR '**strengthening**' OR '**warming up**' OR '**cooling down**' | 1,545,789 |
| #4 | #1 And #2 And #3 | 1,398 |

| **Web of Science** | | |
| --- | --- | --- |
| #1 | **(((((((((TS=(Depression)) OR TS=(Depressive Symptom)) OR TS=(Depressive Symptoms)) OR TS= (Emotional Depression)) OR TS=(Symptom, Depressive)) OR TS=(Depression, Emotional)) OR TS=(Negative Emotion)) OR TS=(Negative Mood)) OR TS=(Affective Disorder)) OR TS=(Affective Disorders)** | **1,097,790**  results |
| #2 | **((((((((((((TS=climacteric women)) OR TS=(menopausal woman)) OR TS=(menopausal women)) OR TS=(menopause females)) OR TS=(menopause women)) OR TS=(climacteric)) OR TS=(menopause feminine) OR TS=(menopause female)) OR TS=(menopausal)) OR TS= (postmenopause)) OR TS=(postmenopausal)) OR TS= (climacteric female))** | **191,341**  results |
| #3 | **((((((((((((((((((((((((((((((((((((((((TS=Exercise)) OR TS=(Exercises)) OR TS=(Physical Activity)) OR TS=(Activities, Physical)) OR TS=(Activity, Physical)) OR TS=(Physical Activities)) OR TS=(Exercise, Physical)) OR TS=(Exercises, Physical)) OR TS=(Physical Exercise)) OR TS=(Physical Exercises)) OR TS=(Acute Exercise)) OR TS=(Acute Exercises)) OR TS=(Exercise, Acute)) OR TS=(Exercises, Acute)) OR TS=(Exercise, Isometric)) OR TS=(Exercises, Isometric)) OR TS=(Isometric Exercises)) OR TS=(Isometric Exercise)) OR TS=(Exercise, Aerobic)) OR TS=(Aerobic Exercise)) OR TS=(Aerobic Exercises)) OR TS=(Exercises, Aerobic)) OR TS=(Exercise Training)) OR TS=(Exercise Trainings)) OR TS=(Training, Exercise)) OR TS=(Trainings, Exercise)) OR TS=(Aerobic)) OR TS=(Jog)) OR TS=(Walk)) OR TS=(Pilates)) OR TS=(Strength training)) OR TS=(Stretching)) OR TS=(Ambulation)) OR TS=(Yoga)) OR TS=(Swim)) OR TS=(Dance)) OR TS=(Dancing)) OR TS=(strengthening)) OR TS=(warming up)) OR TS=(cooling down)** | **3,289,316** |
| #4 | #1 AND #2 And #3 And | 1,196 |

| **Cochrane** | | |
| --- | --- | --- |
| #1 | MeSH descriptor: [Depression] explode all trees | 118550 |
| #2 | (Depression) OR (Depressive Symptom) OR (Depressive Symptoms) OR (Emotional Depression) OR (Symptom, Depressive) | 119473 |
| #3 | (Depression, Emotional) OR (Negative Emotion) OR (Negative Mood) OR (Affective Disorder) OR (Affective Disorders) | 20177 |
| #4 | #1 OR #2 OR #3 | 128604 |
| #5 | MeSH descriptor: [climacteric women] explode all trees | 1624 |
| #6 | (climacteric women) OR (menopausal woman) OR (menopausal women) OR (menopause females) OR (menopause women) | 12475 |
| #7 | (climacteric) OR (menopause feminine) OR (menopause female) OR (menopausal) OR (postmenopause) | 23487 |
| #8 | (postmenopausal) OR (climacteric female) | 24859 |
| #9 | #5 OR #6 OR #7 OR #8 | 32867 |
| #10 | MeSH descriptor: [Exercise] explode all trees | 153527 |
| #11 | (Exercise) OR (Exercises) OR (Physical Activity) OR (Activities, Physical) OR (Activity, Physical) | 200398 |
| #12 | (Physical Activities) OR (Exercise, Physical) OR (Exercises, Physical) OR (Physical Exercise) OR (Physical Exercises) | 74510 |
| #13 | (Acute Exercise) OR (Acute Exercises) OR (Exercise, Acute) OR (Exercises, Acute) OR (Exercise, Isometric) | 18821 |
| #14 | (Exercises, Isometric) OR (Isometric Exercises) OR (Isometric Exercise) OR (Aerobic Exercise) OR (Exercise, Aerobic) | 25414 |
| #15 | (Aerobic Exercises) OR (Exercises, Aerobic) OR (Exercise Training) OR (Exercise Trainings) OR (Training, Exercise) | 54610 |
| #16 | (Trainings, Exercise) OR (Aerobic) OR (Jog) OR (Walk) OR (Pilates) | 45846 |
| #17 | (Strength training) OR (Stretching) OR (Ambulation) OR (Yoga) OR (Swim) | 45582 |
| #18 | (Dance) OR (Dancing) OR (strengthening) OR (warming up) AND (cooling down):ti,ab,kw | 12601 |
| #19 | #10 OR #11 OR #12 OR #13 OR #14 OR #15 OR #16 OR #17 OR #18 | 235282 |
| #20 | #4 and #9 and #19 | 331 |

#

# **Appendix C** Dose-response effects from NMA

#
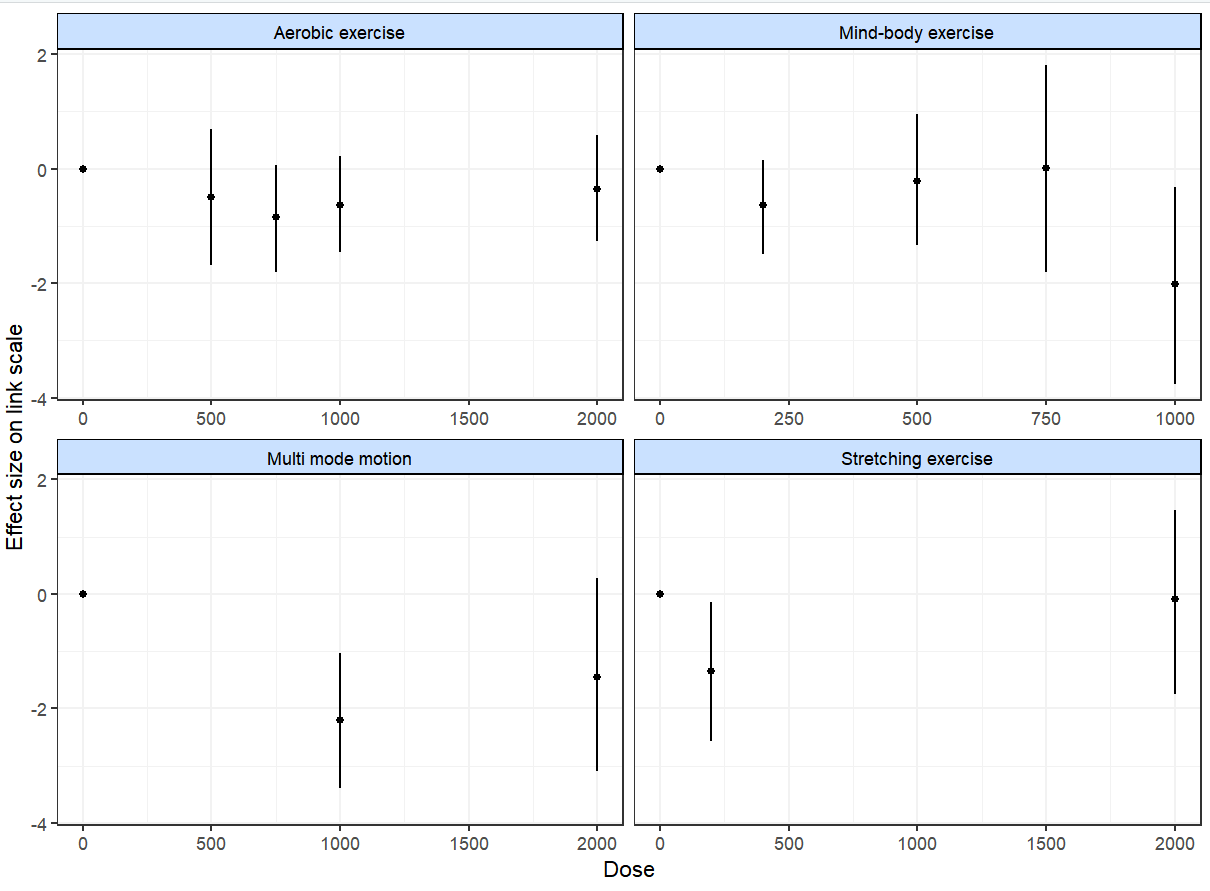


**Appendix D** Quadratic dose–response curves for physical activity interventions estimated from a user-defined polynomial MBNMA model with 95% credible intervals.

#
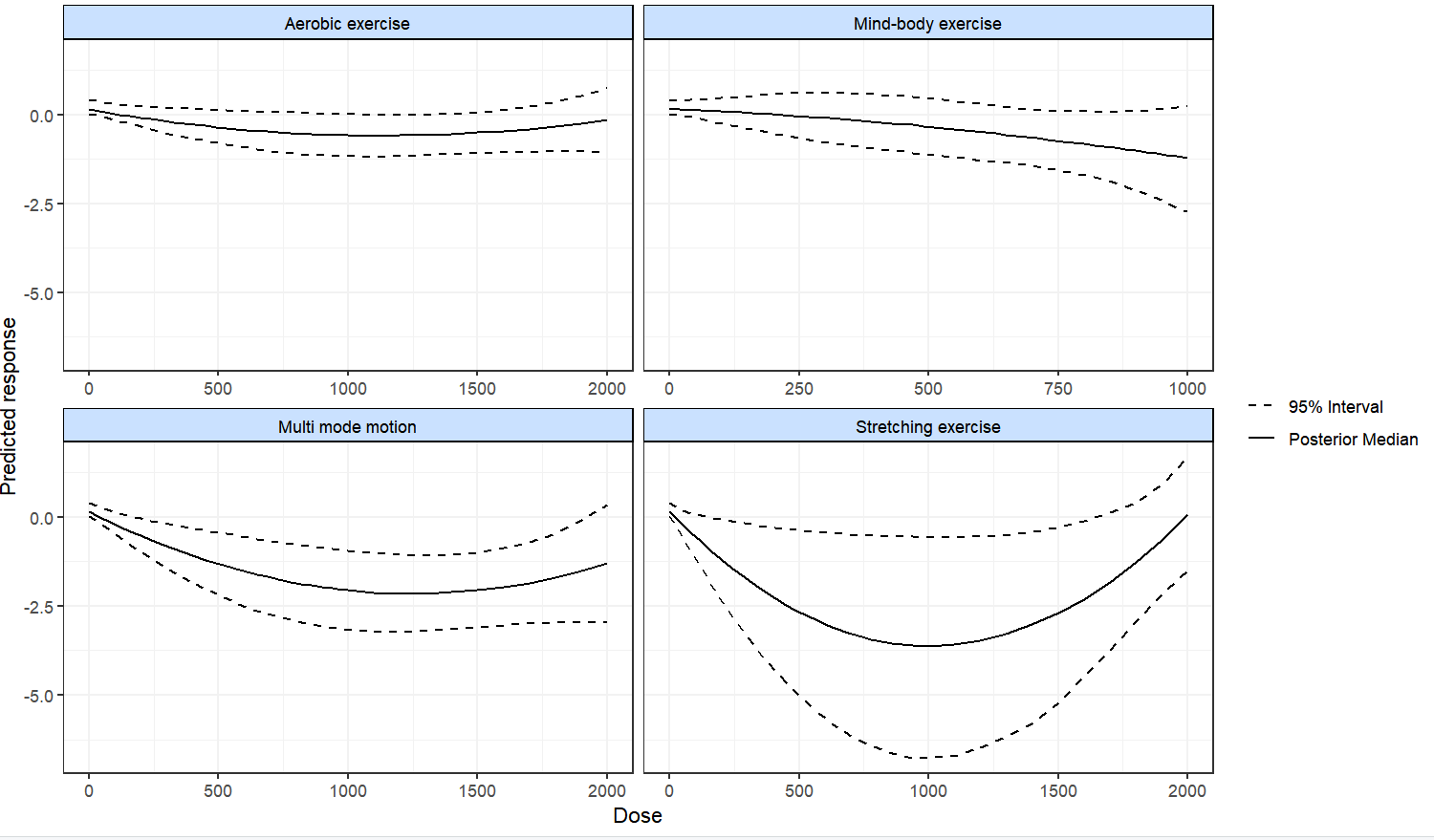


# **Appendix E** Node-Splitting Analysis of Inconsistency in Dose-Response Network

| Comparison | p-value | Direct Effect | 95% CrI (lower) | 95% CrI (upper) | Indirect Effect | Indirect 95% CrI (lower) | Indirect 95% CrI (upper) | MBNMA Effect | MBNMA 95% CrI (lower) | MBNMA 95% CrI (upper) |
| --- | --- | --- | --- | --- | --- | --- | --- | --- | --- | --- |
| Stretching exercise_2000 vs Placebo_0 | 0.048 | -0.084 | -1.868 | 1.696 | -13.647 | -26.845 | -0.467 | -0.294 | -2.133 | 1.618 |
| Stretching exercise_200 vs Placebo_0 | 0.043 | -1.351 | -2.646 | -0.093 | -0.009 | -0.194 | 0.166 | -0.029 | -0.213 | 0.162 |
| Multi mode motion_2000 vs Placebo_0 | 0.176 | -1.481 | -3.351 | 0.376 | -4.436 | -7.185 | -1.909 | -2.496 | -4.159 | -0.891 |
| Multi mode motion_1000 vs Placebo_0 | 0.17 | -2.213 | -3.49 | -1.0 | -0.73 | -1.641 | 0.181 | -1.248 | -2.079 | -0.446 |
| Mind-body exercise_1000 vs Placebo_0 | 0.44 | -2.0 | -4.015 | -0.028 | -0.634 | -2.348 | 1.071 | -1.211 | -2.562 | 0.029 |
| Mind-body exercise_750 vs Placebo_0 | 0.398 | 0.026 | -1.867 | 1.932 | -1.217 | -2.383 | -0.089 | -0.908 | -1.921 | 0.022 |
| Mind-body exercise_500 vs Placebo_0 | 0.552 | -0.206 | -1.641 | 1.173 | -0.742 | -1.507 | 0.026 | -0.605 | -1.281 | 0.015 |
| Mind-body exercise_200 vs Placebo_0 | 0.317 | -0.671 | -1.688 | 0.277 | -0.212 | -0.473 | 0.078 | -0.242 | -0.512 | 0.006 |
| Aerobic exercise_2000 vs Placebo_0 | 0.311 | -0.317 | -1.346 | 0.683 | -1.592 | -3.07 | -0.164 | -0.754 | -1.642 | 0.122 |
| Aerobic exercise_1000 vs Placebo_0 | 0.583 | -0.628 | -1.624 | 0.399 | -0.317 | -0.813 | 0.23 | -0.377 | -0.821 | 0.061 |
| Aerobic exercise_750 vs Placebo_0 | 0.294 | -0.862 | -1.993 | 0.215 | -0.234 | -0.574 | 0.114 | -0.283 | -0.616 | 0.046 |
| Aerobic exercise_500 vs Placebo_0 | 0.292 | -0.515 | -1.907 | 0.832 | -0.181 | -0.421 | 0.061 | -0.188 | -0.411 | 0.031 |

Note: Direct and indirect comparisons were assessed for inconsistency using node-splitting. MBNMA: model-based network meta-analysis estimates. CrI: credible interval.

# **Appendix F** Full UME Model Results

| Parameter | Mean | SD | 2.5% CrI | 25% CrI | 50% CrI | 75% CrI | 97.5% CrI | Rhat | n.eff |
| --- | --- | --- | --- | --- | --- | --- | --- | --- | --- |
| rate[1,1] | 0.0 | 0.0 | 0.0 | 0.0 | 0.0 | 0.0 | 0.0 | 1.0 | 1 |
| rate[2,1] | -0.084 | 0.032 | -0.148 | -0.105 | -0.084 | -0.063 | -0.02 | 1.002 | 1300 |
| rate[3,1] | -0.105 | 0.048 | -0.202 | -0.135 | -0.105 | -0.075 | -0.012 | 1.002 | 1300 |
| rate[4,1] | -0.276 | 0.065 | -0.41 | -0.318 | -0.274 | -0.235 | -0.148 | 1.001 | 2800 |
| rate[5,1] | -0.127 | 0.079 | -0.277 | -0.182 | -0.126 | -0.076 | 0.031 | 1.001 | 3000 |
| rate[2,2] | 0.0 | 0.0 | 0.0 | 0.0 | 0.0 | 0.0 | 0.0 | 1.0 | 1 |
| rate[3,2] | 1.032 | 31.856 | -60.646 | -20.488 | 1.431 | 22.178 | 62.585 | 1.001 | 3000 |
| rate[4,2] | 0.234 | 31.821 | -59.868 | -21.593 | -0.572 | 22.146 | 63.354 | 1.001 | 3000 |
| rate[5,2] | -0.871 | 32.123 | -65.076 | -22.262 | -1.336 | 20.775 | 62.57 | 1.001 | 3000 |
| rate[3,3] | 0.0 | 0.0 | 0.0 | 0.0 | 0.0 | 0.0 | 0.0 | 1.0 | 1 |
| rate[4,3] | -0.455 | 31.431 | -61.615 | -21.108 | -1.426 | 20.989 | 61.655 | 1.001 | 3000 |
| rate[5,3] | -1.119 | 31.427 | -64.397 | -21.809 | -0.891 | 20.32 | 60.013 | 1.002 | 1200 |
| rate[4,4] | 0.0 | 0.0 | 0.0 | 0.0 | 0.0 | 0.0 | 0.0 | 1.0 | 1 |
| rate[5,4] | -0.092 | 31.989 | -63.09 | -22.31 | 0.063 | 21.403 | 63.913 | 1.001 | 3000 |
| rate[5,5] | 0.0 | 0.0 | 0.0 | 0.0 | 0.0 | 0.0 | 0.0 | 1.0 | 1 |
| sd | 0.746 | 0.143 | 0.516 | 0.643 | 0.728 | 0.827 | 1.072 | 1.001 | 3000 |
| totresdev | 53.347 | 10.563 | 34.738 | 45.734 | 52.675 | 60.348 | 75.566 | 1.001 | 3000 |
| deviance | 72.485 | 10.563 | 53.876 | 64.872 | 71.813 | 79.486 | 94.704 | 1.001 | 3000 |

# Table S1 Dose–Response Effects of Exercise Modalities on Menopausal Depression

| Intervention | Dose (METs/week) | SMD | 95% CrI (lower) | 95% CrI (upper) |
| --- | --- | --- | --- | --- |
| Placebo | 0 | 0.1699 | 0.0236 | 0.4141 |
| Aerobic exercise | 220 | -0.0974 | -0.3800 | 0.2239 |
| Aerobic exercise | 670 | -0.4647 | -1.0021 | 0.0636 |
| Aerobic exercise | 1100 | -0.5907 | -1.1847 | -0.0139 |
| Mind-body exercise | 330 | -0.1192 | -0.8352 | 0.6284 |
| Mind-body exercise | 780 | -0.7806 | -1.6634 | 0.0955 |
| Mind-body exercise | 1000 | -1.2161 | -2.6900 | 0.2861 |
| Multi mode motion | 220 | -0.5757 | -1.0847 | -0.0803 |
| Multi mode motion | 670 | -1.6524 | -2.6913 | -0.6529 |
| Multi mode motion | 1300 | -2.1476 | -3.1890 | -1.0687 |
| Stretching exercise | 220 | -1.3105 | -2.5660 | -0.1049 |
| Stretching exercise | 670 | -3.2063 | -6.0242 | -0.4645 |
| Stretching exercise | 1300 | -3.3051 | -6.1722 | -0.5043 |

Note: Estimates are standardized mean differences (SMDs) and 95% credible intervals (CrIs) from a Bayesian quadratic dose–response model. Negative values indicate reductions in depressive symptoms (lower is better). CrI crosses 0, indicating a non-significant effect.

# Table S2 Results of Network Meta-Analysis

| Parameter | Treatment (Dose) | Mean (SMD) | SD | 2.5% CrI | 50% CrI | 97.5% CrI | Rhat | n.eff |
| --- | --- | --- | --- | --- | --- | --- | --- | --- |
| d[1] | Aerobic exercise (500mg) | 0.0 | 0.0 | 0.0 | 0.0 | 0.0 | 1.0 | 1 |
| d[2] | Aerobic exercise (750mg) | -0.512 | 0.593 | -1.687 | -0.5 | 0.701 | 1.001 | 3000 |
| d[3] | Aerobic exercise (1000mg) | -0.857 | 0.472 | -1.806 | -0.85 | 0.065 | 1.001 | 3000 |
| d[4] | Aerobic exercise (2000mg) | -0.632 | 0.423 | -1.451 | -0.642 | 0.213 | 1.001 | 3000 |
| d[5] | Mind-body exercise (200mg) | -0.35 | 0.466 | -1.27 | -0.352 | 0.587 | 1.001 | 3000 |
| d[6] | Mind-body exercise (500mg) | -0.646 | 0.423 | -1.494 | -0.633 | 0.157 | 1.001 | 3000 |
| d[7] | Mind-body exercise (750mg) | -0.2 | 0.574 | -1.326 | -0.21 | 0.948 | 1.001 | 3000 |
| d[8] | Mind-body exercise (1000mg) | -0.001 | 0.892 | -1.809 | 0.007 | 1.811 | 1.001 | 3000 |
| d[9] | Multi mode motion (1000mg) | -2.025 | 0.853 | -3.76 | -2.019 | -0.328 | 1.002 | 1300 |
| d[10] | Multi mode motion (2000mg) | -2.213 | 0.586 | -3.385 | -2.209 | -1.037 | 1.003 | 710 |
| d[11] | Placebo (0mg) | -1.447 | 0.856 | -3.085 | -1.449 | 0.277 | 1.001 | 3000 |
| d[12] | Stretching exercise (200mg) | -1.351 | 0.613 | -2.573 | -1.343 | -0.143 | 1.001 | 3000 |
| d[13] | Stretching exercise (2000mg) | -0.096 | 0.826 | -1.752 | -0.081 | 1.466 | 1.001 | 3000 |

# Figure S1 Posterior Distributions of Spline Parameters (β₁, β₂) by Exercise Type in Nonparametric MBNMA


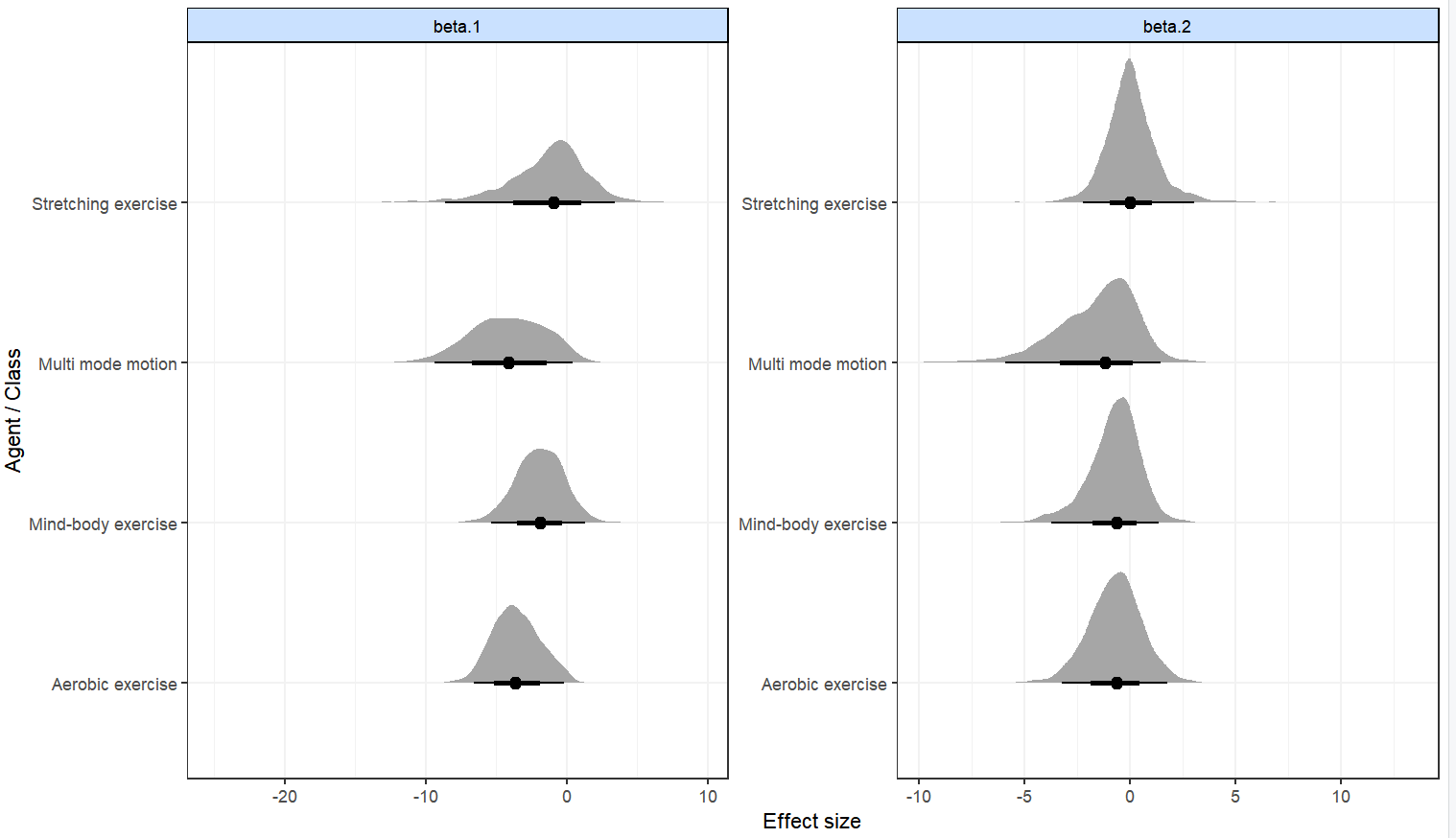


**Figure S2**  NMA-estimated dose–response curve for physical activity interventions.


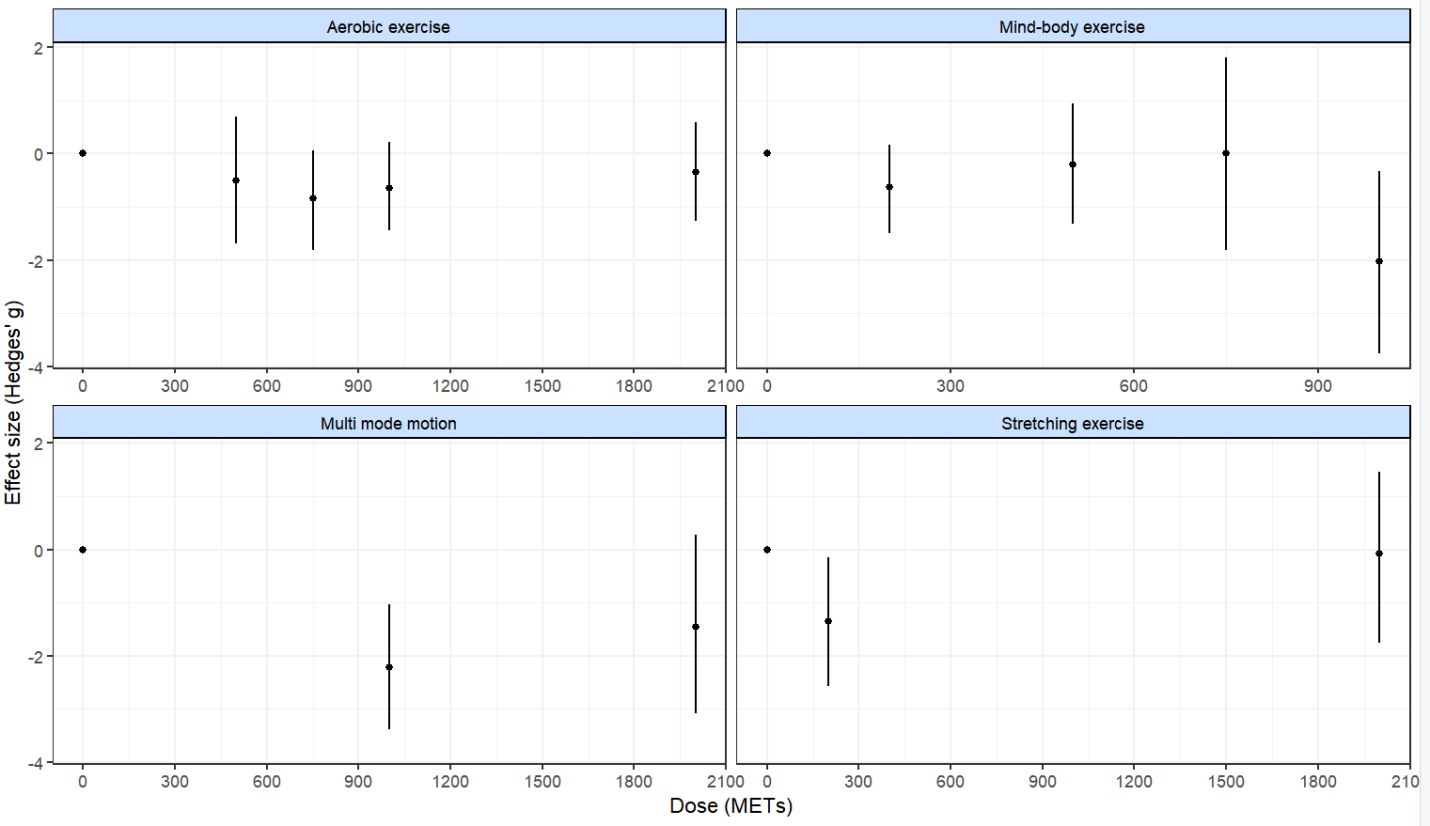


**FIgure S3** RCS-Estimated Dose–Response Curves for Exercise Interventions: Solid Lines Denote Posterior Medians; Dashed Lines 95% Credible Intervals


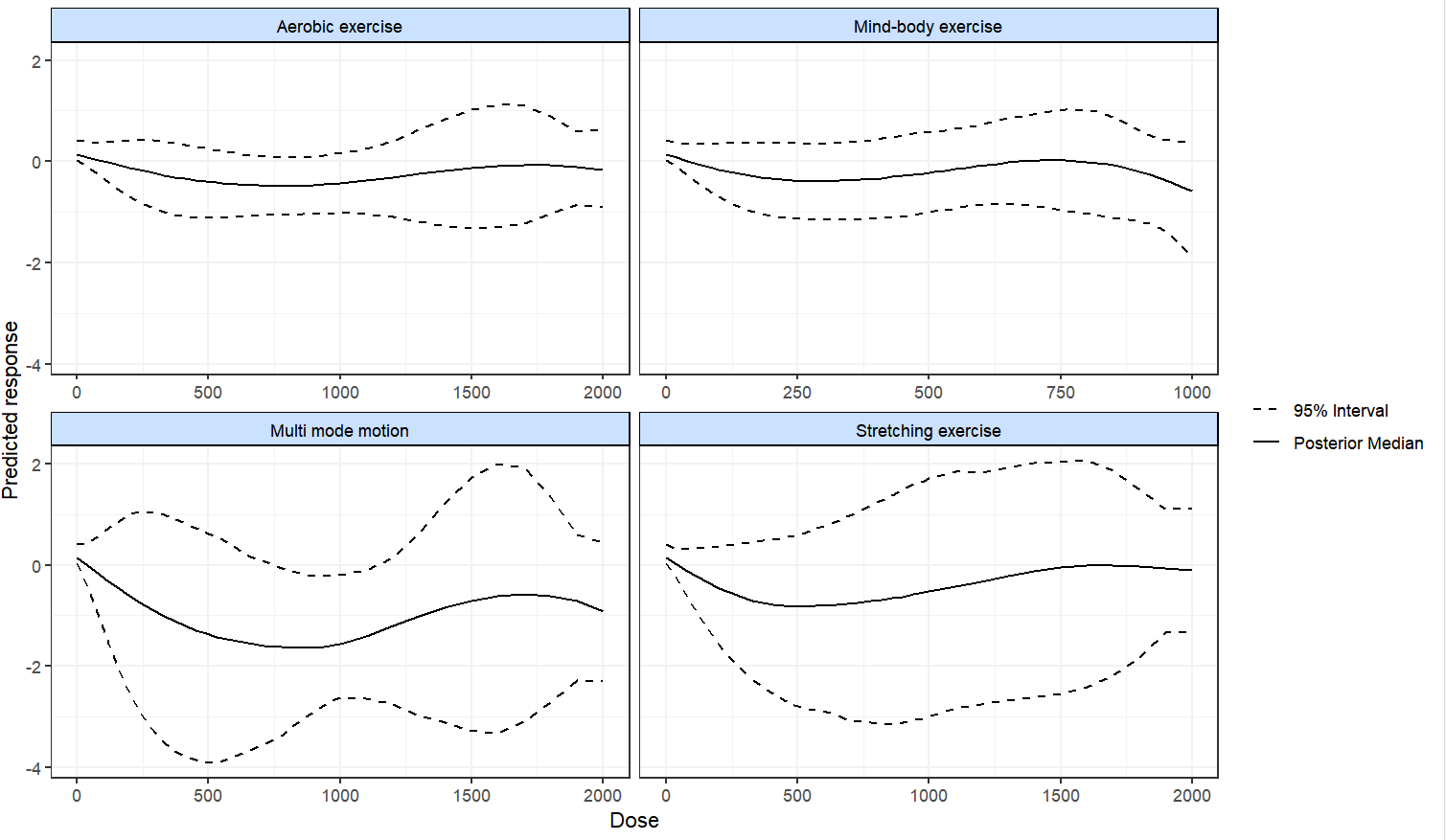

Supplement: Supplementary file 1 [file Table_1.DOCX]
